# mDia formins form hetero-oligomers and cooperatively maintain murine hematopoiesis

**DOI:** 10.1371/journal.pgen.1011084

**Published:** 2023-12-29

**Authors:** Zhaofeng Li, Meng Su, Xinshu Xie, Pan Wang, Honghao Bi, Ermin Li, Kehan Ren, Lili Dong, Zhiyi Lv, Xuezhen Ma, Yijie Liu, Baobing Zhao, Yuanliang Peng, Jing Liu, Lu Liu, Jing Yang, Peng Ji, Yang Mei

**Affiliations:** 1 Hunan Provincial Key Laboratory of Animal Model and Molecular Medicine, Hunan University, Changsha, China; 2 School of Biomedical Sciences, Hunan University, Changsha, China; 3 Department of Pathology, Feinberg School of Medicine, Northwestern University, Chicago, Illinois, United States of America; 4 Department of Pharmacology, School of Pharmaceutical Sciences, Cheeloo College of Medicine, Shandong University, Jinan, Shandong, China; 5 Department of Hematology, the Second Xiangya Hospital; Molecular Biology Research Center, School of Life Sciences; Hunan Province Key Laboratory of Basic and Applied Hematology, Central South University; Changsha, China; Centre for Cancer Biology, SA Pathology, AUSTRALIA

## Abstract

mDia formin proteins regulate the dynamics and organization of the cytoskeleton through their linear actin nucleation and polymerization activities. We previously showed that mDia1 deficiency leads to aberrant innate immune activation and induces myelodysplasia in a mouse model, and mDia2 regulates enucleation and cytokinesis of erythroblasts and the engraftment of hematopoietic stem and progenitor cells (HSPCs). However, whether and how mDia formins interplay and regulate hematopoiesis under physiological and stress conditions remains unknown. Here, we found that both mDia1 and mDia2 are required for HSPC regeneration under stress, such as serial plating, aging, and reconstitution after myeloid ablation. We showed that mDia1 and mDia2 form hetero-oligomers through the interactions between mDia1 GBD-DID and mDia2 DAD domains. Double knockout of mDia1 and mDia2 in hematopoietic cells synergistically impaired the filamentous actin network and serum response factor-involved transcriptional signaling, which led to declined HSPCs, severe anemia, and significant mortality in neonates and newborn mice. Our data demonstrate the potential roles of mDia hetero-oligomerization and their non-rodent functions in the regulation of HSPCs activity and orchestration of hematopoiesis.

## Author summary

Blood production and generation entirely depend on the hematopoietic stem progenitor cells (HSPCs) that have to be tightly controlled. mDia formin proteins are critical regulators for linear-actin synthesis that are involved in actin filament formation and cytoskeleton organization. Previous studies have revealed the important roles of mDia1 or mDia2 in regulating the functions of T cells, neutrophils, or red blood cells, but there is a lack of evidence for the non-redundant roles of these two factors *in vivo*. Here we combined genetic manipulation of the mDia1 and mDia2 double deficient mouse with biochemical analysis. We find that mDia1 interacts directly with mDia2 to form hetero-oligomers *in vitro* and *in vivo*, and both mDia1 and mDia2 are required to maintain HSPCs hemostasis during stress in mice. Dual deficiency of mDia1 and mDia2 in mice leads to compromised serum response factor (SRF) signaling, with declined HSPC, severe anemia, and increased animal mortality. Our work not only provides a previously unrecognized working model for the mDia1-mDia2 complex but also highlights their cooperative roles in hematopoiesis.

## Introduction

Hematopoiesis is a continuous and dynamic process initiated by hematopoietic stem and progenitor cells (HSPCs). The proper maintenance and regulation of HSPC functions ensure the downstream differentiation of various hematopoietic lineages. Dysregulated HSPC differentiation and self-renewal compromise the integrity of hematopoietic homeostasis, which could lead to bone marrow failure or hematologic malignancies [1, 2]. One of the well-known regulators of HSPC functions is the actin cytoskeleton network. The establishment and maintenance of cell polarization, adhesion, and motility are tightly regulated by the intracellular actin cytoskeleton, which is particularly important for the diverse functions of hematopoietic cells. The cellular actin network is strictly dependent on the synthesis of actin filaments (F-actin), a process that requires the involvement of actin polymerization. In mammals, two major actin-nucleating protein families exist including the Wiskott–Aldrich syndrome protein (WASP)-Arp2/3 protein complexes and formin proteins [3–7]. In contrast to the branched F-actin filaments formation by WASP-Arp2/3 complex, formin proteins facilitate the linear actin polymerization [4, 8, 9]. The formin subfamily diaphanous-related formins (mDia) include mDia1, mDia2, and mDia3 [10]. mDia formins are highly conserved and characterized by the unique actin polymerization formin homology domains (FH1 and FH2). By binding with profilin, FH domains sequester free monomeric G-actin to initiate the synthesis and elongation of linear F-actin [9, 11–13]. The N-terminus of mDia formins is composed of a Rho GTPase-binding domain (GBD) and a diaphanous inhibitory domain (DID). The C-terminus harbors a diaphanous auto-regulatory domain (DAD). In the inactive form, the DID and DAD domains interact and form an inter-molecular autoinhibitory conformation, which can be disrupted by the binding of Rho GTPase to the GBD domain [4, 9]. Loss of either DID or DAD enables the constitutive activation of mDia formins [12].

mDia formins, specifically mDia1 and mDia2, are known to regulate the development and functions of different blood lineages. For instance, mDia1 regulates T cell trafficking and T cell receptor (TCR) activation [13–15]. It also controls cell adhesion, migration, and activation of dendritic cells and T cells [16–18]. In macrophages, mDia1 is involved in complement receptor (CR3)-mediated phagocytosis[19, 20]. mDia1 also coordinates mast cell migration and granule secretion [21]. Moreover, mDia1 deficient mice exhibit dysplastic granulocytes and neutropenia, which mimics human myelodysplastic syndromes (MDS) [18, 22]. Although mDia1 null mice showed no obvious defects in platelet counts [22], mDia1 knockdown in human CD34^+^ cells perturbed *in vitro* proplatelet formation [10]. The functions of mDia1 in HSPCs are largely unknown. Previously, we and others demonstrated that mDia2 facilitates cytokinesis and enucleation in erythroblasts [23–25]. We also found that mDia2 is required for HSPC lodgment and engraftment in bone marrow during transplantation [3]. Whether mDia formins have redundant or unique roles *in vivo* during hematopoiesis remains elusive.

In this study, we found that both mDia1 and mDia2 are necessary for HSPC regeneration *in vitro* and *in vivo*. Interestingly, mDia1 and mDia2 form hetero-oligomers that lead to an augmented transcriptional activity of serum response factor (SRF). Dual deficiency of mDia1 and mDia2 led to declined HSPCs, severe anemia in neonates, and increased death in newborn mice. mDia1/2 double null HSPCs also exhibited compromised gene expression signature of SRF signaling. These studies suggest the potential roles of mDia hetero-oligomerization in the regulation of murine hematopoiesis.

## Results

### mDia1 is dispensable for the composition of HSPCs under steady state

**Fig 1:**
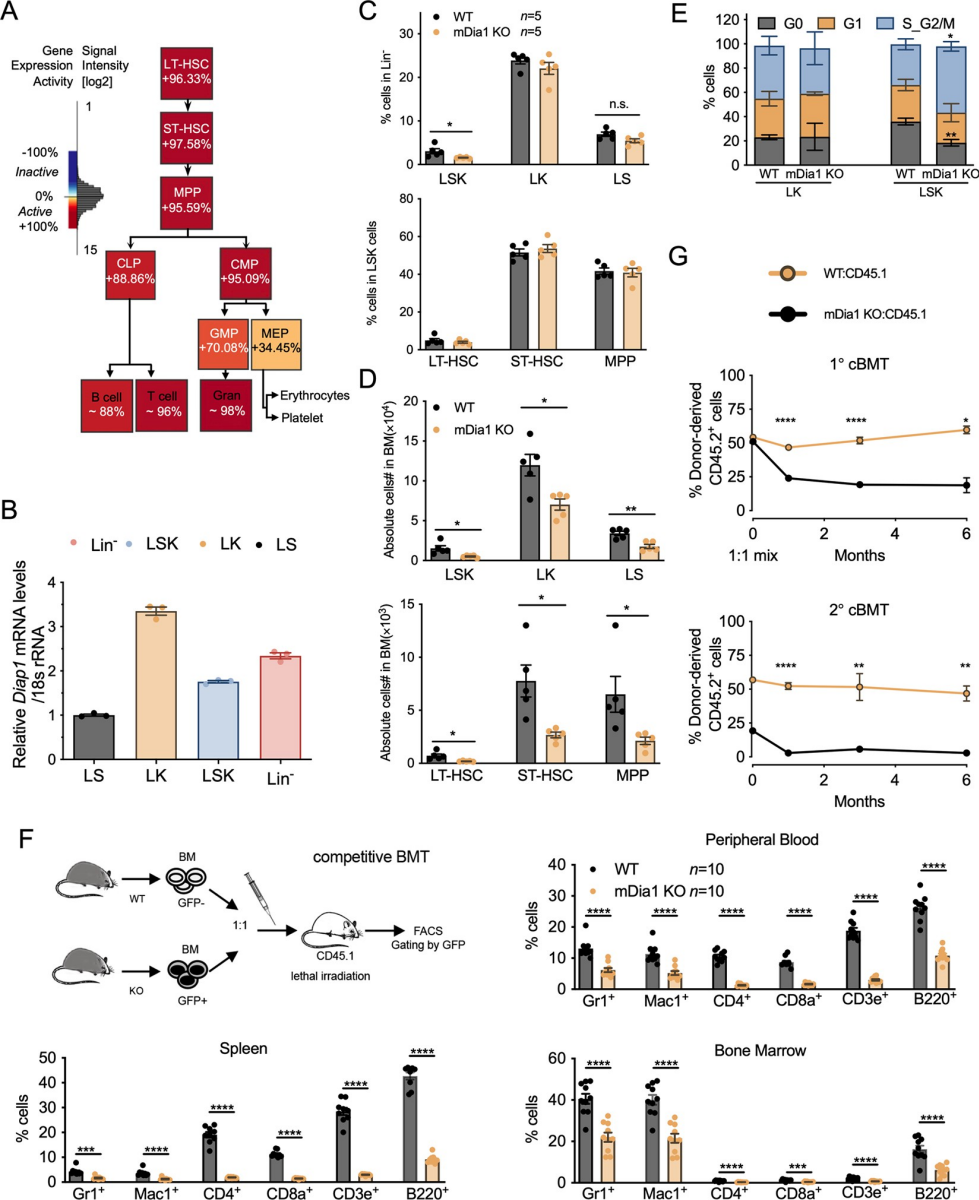
mDia1 regulates HSPC functions in bone marrow transplantation. (A). mDia1 expression pattern in murine hematopoietic cells obtained from Gene Expression Commons database. *(B)*. *Diap1* transcript levels in the indicated sorted cell lineages were examined by quantitative real-time PCR analyses. 18S ribosomal RNA was used as an internal control. The experiment was repeated in triplicate. (C). Quantification of the flow cytometric analyses on the percentages of the indicated HSPC populations in the bone marrow of recipient mice 3 months post-transplantation with wild type (WT) or mDia1 knockout (mDia1 KO) BMMCs (2×10^6^). (D). Same as C except the absolute cell numbers were quantified. (E). Quantitative analyses of different cell cycle stages of the indicated bone marrow HSPCs from the transplanted mice receiving WT or mDia1 KO cells in C. (F). Chimerism analyses of the competitive transplantation assay in the indicated tissues by tracking mDia1 KO cells harboring intrinsic GFP expression. Data were obtained 2 months post-transplantation. (G). Chimerism analyses of primary (upper panel) and secondary (lower panel) competitive transplantation assays were performed to monitor the long-term engraftment of mDia1 WT and KO HSPCs. Total bone marrow cells from the recipients in the primary transplantation assays were used for the secondary transplantation. Error bars represent the SEM of the mean. * *p*<0.05, ** *p*<0.01, *** *p*<0.001, **** *p*<0.0001, n.s., not significant. Two-tailed unpaired student’s t-test was used to generate the *p* values.

mDia formins exhibit distinct expression profiles in mice. In hematopoietic cells, mDia1 and mDia2 are highly expressed whereas mDia3 has only been reported in T cells [14, 24, 26]. Our previous studies demonstrated that mDia2 is essential for the HSPC lodgment to the bone marrow niche during transplantation [3]. Similar to mDia2, mDia1 is also highly enriched in all the hematopoietic lineages, including HSPCs, as analyzed by the Gene Expression Commons database [27] (Fig 1A). This is further confirmed through a quantitative real-time PCR assay where we detected a considerable level of *Diap1* (encoding mDia1) transcript in HSPCs, particularly in lineage negative, c-Kit^+^ (LK) cells (Figs 1B and S1A). To examine the role of mDia1 in HSPC functions *in vivo*, we first analyzed HSPC contents in mDia1 whole body knockout (KO) mice (8–10 weeks old), including LSK, LK, LT-HSC (long-term HSCs, CD34-CD135-LSK), ST-HSC (short-term HSCs, CD34+CD135-LSK) and MPP (multipotent progenitors, CD34+CD135+LSK), as we previously performed [3, 22, 28] (S1A Fig). mDia1 deficient HSPCs in the bone marrow showed no detectable differences in number compared to their wild-type (WT) littermate counterparts (S1B and S1C Fig). These results indicate that mDia1 is probably dispensable for HSPC functions under the steady state in young adult mice.

### mDia1 is required for HSPC homeostasis during transplantation and aging

To test the functions of mDia1 in HSPCs under stress conditions, we performed a bone marrow transplantation assay using bone marrow mononuclear cells (BMMCs) from mDia1 KO mice. Recipient mice transplanted with BMMCs from 12-week-old mDia1 KO mice exhibited decreased cell counts of LSK, LT-HSC, ST-HSC, and MPP populations (Fig 1C and 1D), and loss of quiescence in LSK populations (Fig 1E). We next performed a competitive transplantation assay in which an equal amount of BMMCs from mDia1 KO and WT littermate control mice (CD45.2+) were transplanted into lethally irradiated recipient mice (CD45.1+). Taking the advantage of enhanced green fluorescent protein (GFP) expression driven by *Diap1* promoter during the construction of mDia1 KO mice[29], mDia1 KO HSPCs can be distinguished from the WT competitors by GFP positive gating in this assay (Fig 1F, upper left panel). Chimerism analyses of peripheral blood, spleen, and bone marrow from the recipient mice revealed that mDia1 deficient hematopoietic lineages were significantly outcompeted by their WT counterparts (Fig 1F). This result has also been verified by using CD45.1+ congenic mice as the competitor cells. Consistently, mDia1 KO cells engrafted inefficiently in recipients when competing with the wildtype CD45.1+ counterparts over time (Fig 1G, upper panel). More importantly, mDia1 deficient donor cells completely declined after a secondary transplantation (Fig 1G, lower panel). By contrast, when examined by *in vivo* homing assay, mDia1 KO cells exhibited similar migration capacity into the bone marrow in a short period during transplantation (S2A–S2C Fig). Altogether, these results indicate that mDia1 is critical for the long-term repopulation of HSPCs during serial bone marrow transplantation.

**Fig 2:**
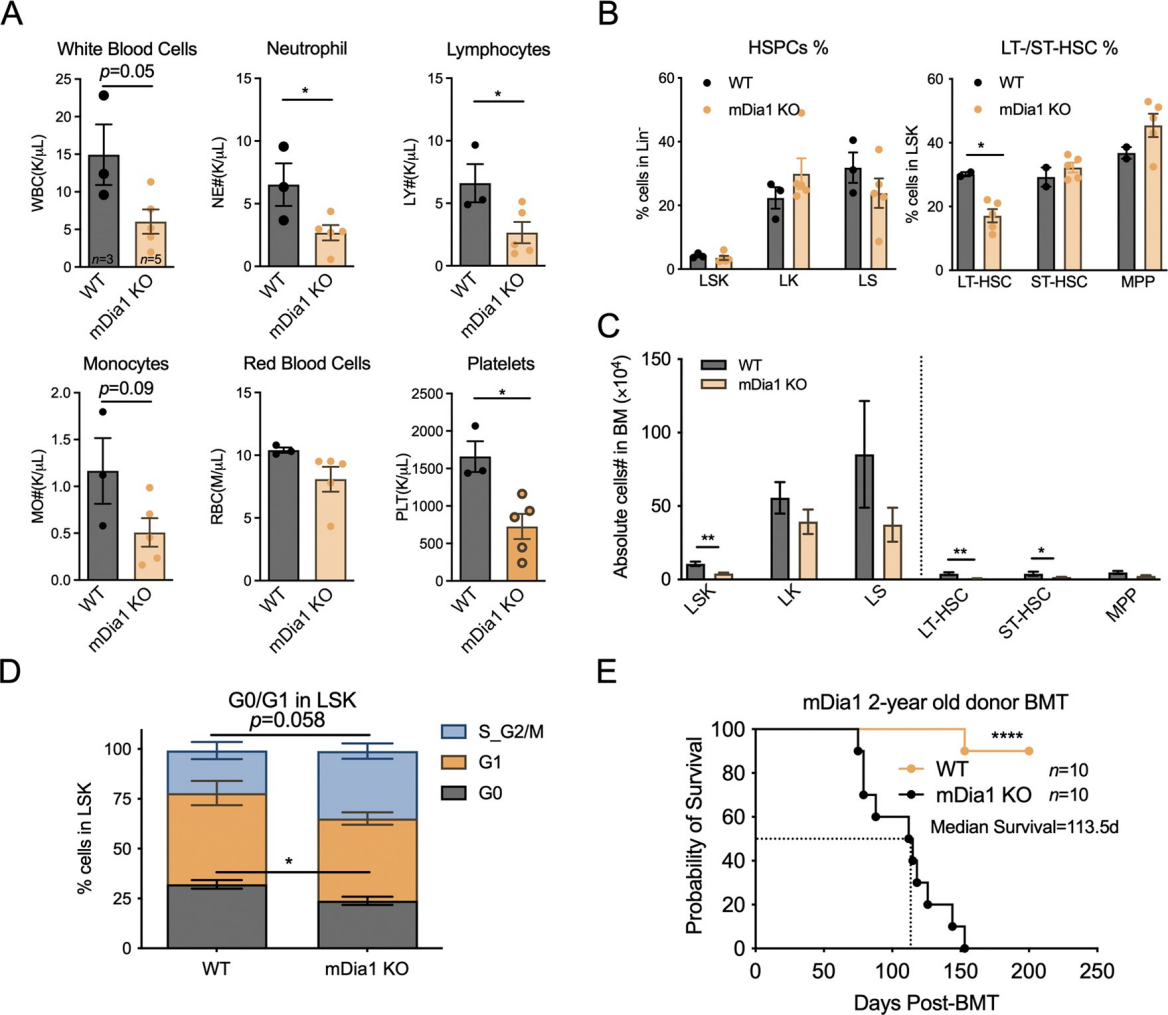
mDia1 is required for HSPC homeostasis during aging. (A) Peripheral blood cell counts in 2-year-old WT and mDia1 KO mice. (B-C) Flow cytometric analyses of the indicated HSPC subpopulations in mice from A. The percentage and total cell number were scored and illustrated in B and C, respectively. (D) Cell cycle profiling of bone marrow LSK cells from mice in A. (E) Kaplan-Meier survival analysis of the recipient mice transplanted with 2-years old WT and mDia1 KO BMMC (2 × 10^6^ per mouse). Error bars represent the SEM of the mean. * *p*<0.05, ** *p*<0.01, *** *p*<0.001 and **** *p*<0.0001. All the *p* values were generated by a two-tailed unpaired student’s t-test, except the p-value in E was calculated by the Mantel-Cox log-rank test.

Aging leads to a gradual loss of self-renewal and regenerative potentials of stem cells and is another stress condition to examine the integrity of HSPCs. Although the epigenomic profiling by deep-sequencing revealed no significant changes in *Diap1* expression in young and aged HSCs[30](S3A and S3B Fig), we observed significant pan-cytopenia in two-year-old mDia1 knockout mice with decreased LT-HSC, ST-HSC, LSK, and LS populations (Fig 2A–2C). Moreover, old mDia1 LSK cells were less quiescent as evidenced by the decrease in G0 and increase in the S/G2-M phase of the cell cycle (Fig 2D). We then performed a bone marrow transplantation assay in which 2-year-old BMMCs from mDia1 deficient mice were transplanted into lethally irradiated young recipient mice (CD45.1+). Compared to the recipient mice transplanted with BMMCs from age-matched WT control mice, mice transplanted with old mDia1 deficient BMMCs showed a rapid lethality with a mean survival of 113.5 days (Fig 2E). Taken together, these data suggest that mDia1 plays a pivotal role in maintaining HSPC homeostasis during transplantation and aging.

### mDia2 is required for HSPC regeneration during stress

Given the highly conserved functions of mDia formins and the key roles of mDia2 in HSPCs lodgment, we reasoned that mDia2 could also contribute to the regeneration of HSPCs under stress conditions. To test this hypothesis, we first assessed the HSPCs compositions in two-year-old mDia2 conditional knockout mice (mDia2^fl/fl^ Vav-Cre). Except for the expected anemia, mDia2 deficient mice did not exhibit other cytopenias in peripheral blood or declined HSPCs in bone marrow (S4A and S4B Fig). In contrast, mild expansion of LSK, ST-HSC, and MPP was observed in these old mice (S4B Fig). The quiescent status of LSK cells was not altered either after mDia2 depletion (S4C Fig).

**Fig 3:**
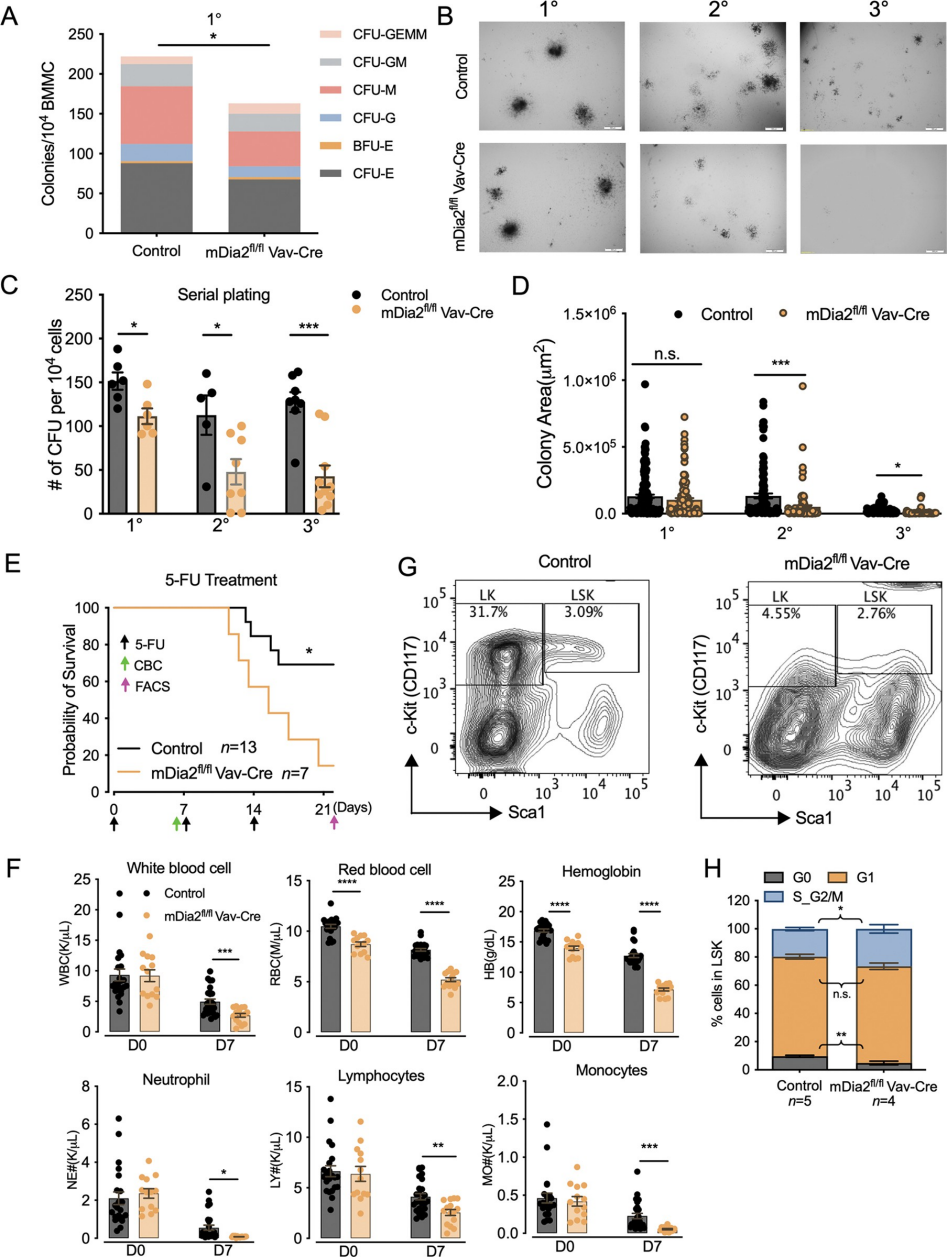
mDia2 regulates stress-induced hematopoietic cell re-population. (A) The composition of indicated colony forming units (CFU) determined based on morphology from primary plate seeded with 1×10^4^ BMMC cells from control or mDia2^fl/fl^ Vav-Cre mice. Data are presented as mean ± SEM, *n* = 2 in each group. (B) *In vitro* colony assays with serial plating. Representative phase contract views illustrating colony morphology under primary (1°), secondary (2°), and tertiary (3°) plating. (C) The CFU numbers in B were scored under a microscope. (D) The size of colonies from B was quantified by measuring the area of each unit with Image J. 1°: 162 colonies in control; 130 colonies in mDia2^fl/fl^ Vav-Cre; 2°:109 colonies in control; 99 colonies in mDia2^fl/fl^ Vav-Cre; 3°: 85 colonies in control; 40 colonies in mDia2^fl/fl^ Vav-Cre. Data were pooled from 3 mice in each group for C and D. (E) Kaplan-Meier survival curve depicting the survival of indicated mice challenged three times with 150 mg/kg 5-FU each time. The arrows indicate the experimental timelines for CBC and FACS assays. (F) Peripheral blood cell counts of mice in E determined on day 7. (G) Representative flow cytometric plots showing HSPC populations on day 21 after 5-FU treatment. (H) Cell cycle profiling of HSPCs from G. Error bars represent the SEM of the mean. * *p*<0.05, ** *p*<0.01, *** *p*<0.001, **** *p*<0.0001, n.s., not significant. Two-tailed unpaired student’s t-test was used to generate the *p* values.

We then evaluated the repopulation capacity of mDia2 deficient HSPCs using an *in vitro* colony-forming cell (CFC) assay in which mDia2^fl/fl^ Vav-Cre or littermate control BMMCs (1 × 10^4^ cells) were seeded in semi-solid methylcellulose culture media supplemented with stem-cell factor, IL-3, IL-6, and erythropoietin. Indeed, mDia2 deficient BMMCs generated lower numbers of colonies than their WT counterparts (Fig 3A). Colony morphologic analyses revealed that mDia2 deficiency led to significantly reduced frequencies in progenitors (Fig 3A). More strikingly, when we performed secondary and tertiary CFC assays every 14 days to assess their capacities to maintain progenitor activities over time, we found that mDia2 deficient HSPCs significantly reduced the ability to maintain colony number and size compared with the control cells, indicating that mDia2 is essential for HSPC re-seeding during serial plating *in vitro* (Fig 3B–3D). Similarly, mDia1 knockout bone marrow cells also showed defects in serial plating *in vitro* (S4D and S4E Fig).

We have previously shown that mDia2 deficient HSPCs were outcompeted by their wild-type counterparts during a competitive transplantation assay, mainly due to the failed trans-endothelial migration of mDia2 deficient cells [3]. To test how mDia2 is involved in the *in-situ* replenishing of the hematopoietic compartment following a myelotoxic challenge, we treated the mDia2^fl/fl^ Vav-Cre or littermate control mice with a myelosuppressive chemotherapeutic drug, 5-fluorouracil (5-FU), at 150 mg/kg every week for three doses. As expected, mDia2^fl/fl^ Vav-Cre mice showed more rapid lethality than the control mice (Fig 3E). One week post the first dose of the 5-FU challenge, mDia2 KO mice showed more severe pancytopenia (Fig 3F), correlating with their short survival. We further performed a flow cytometric analysis of HSPCs in those mice that survived after 3 weeks. As expected, mDia2 deficient mice generated significantly fewer cells from LT-HSC to diverse progenitor cells after serial 5-FU insults (Figs 3G and S5A). This defect in bone marrow regeneration was also associated with compromised stem cell quiescence and declined survival (Figs 3H and S5B). Thus, mDia2 protects mice from chemotherapy-induced myelosuppression. Similarly, mDia1 knock-out mice were also susceptible to the 5-FU challenge with shorter survival and pancytopenia (S5C and S5D Fig). These data collectively suggest that mDia formins are essential for hematopoietic recovery from stress-induced HSPC depletion *in vitro* and *in vivo*.

### mDia1 interacts with mDia2 to form a hetero-oligomers

**Fig 4:**
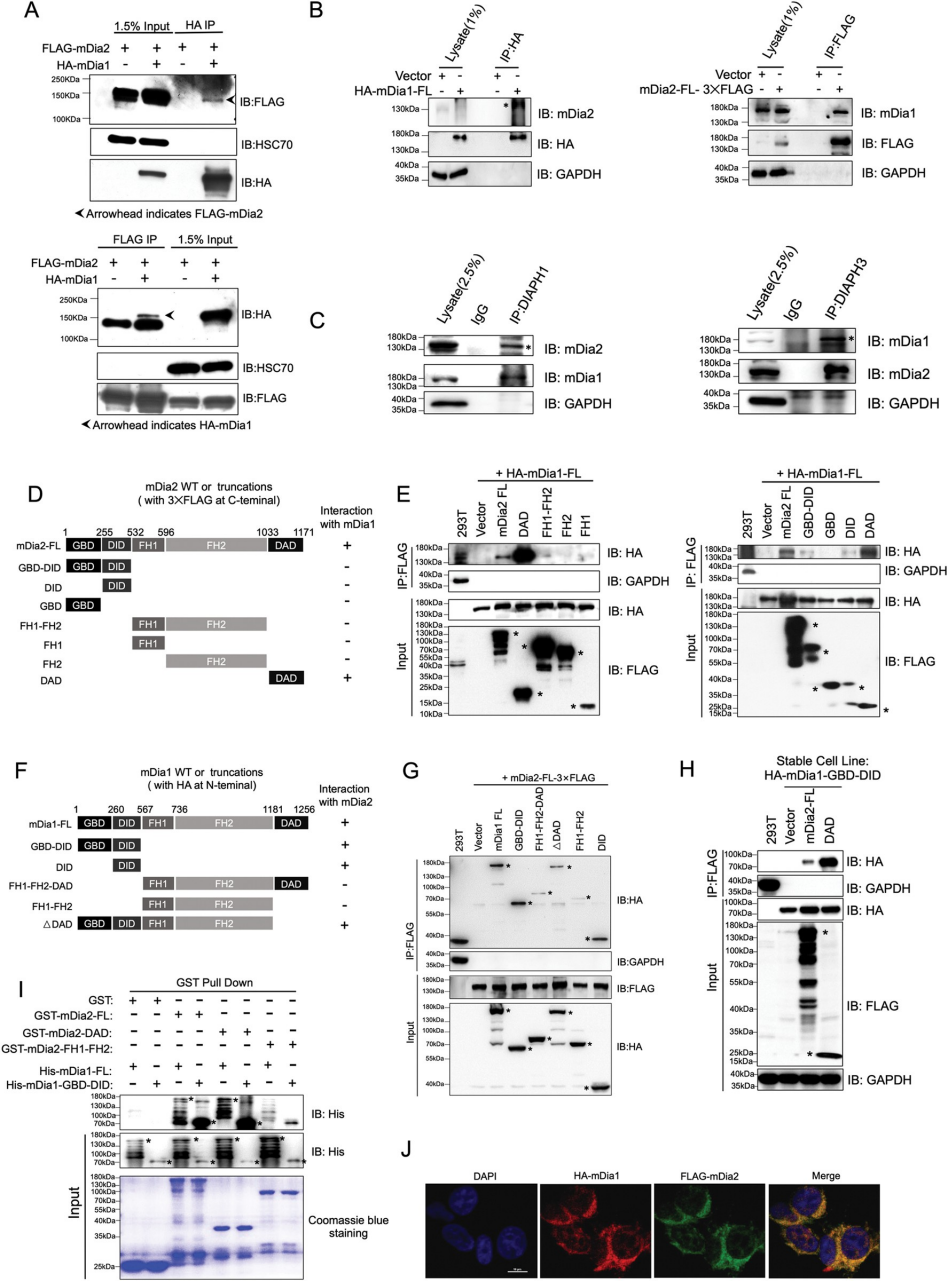
mDia1 and mDia2 form hetero-oligomers. (A) 293T cells were transiently transfected with constructs expressing FLAG-mDia2 with or without HA-mDia1. The cell lysates were collected and immunoprecipitated with anti-HA (upper panel) or anti-FLAG (lower panel) magnetic microbeads. The immunoprecipitants were subjected to Western blotting assays with the indicated antibodies. (B) Immunoprecipitation assay in 293T cells overexpressing HA-mDia1 (left) or mDia2-3×FLAG (right). Western blotting analyses with indicated antibodies following the indicated co-immunoprecipitation assays were shown. (C) Co-immunoprecipitation assays were performed using anti-mDia1 (left) or anti-mDia2 (right) antibodies in 293T cell lysates followed by Western blotting of the indicated proteins. (D) Schematic map showing wild-type mDia2 domains and different truncated mutants. (E) Co-immunoprecipitation of HA-tagged mDia1 full length with indicated mDia2 full length or truncated mutants tagged by C-terminal 3 ×FLAG. The lysates were extracted from 293T cells transfected with indicated constructs, and immunoprecipitation assays were performed using anti-FLAG magnetic beads. The retained proteins on the beads were blotted and visualized by indicated antibodies. (F) Schematic diagram of wild-type mDia1 domains and different truncated mutants. (G) Co-immunoprecipitation of mDia2-3×FLAG with HA-tagged mDia1 full-length or truncated mutants by using anti-HA magnetic beads. The interacted proteins remaining on the beads were examined by Western blotting using indicated antibodies. (H) 293T cells stably expressing HA-mDia1 were transfected with empty vectors or constructs encoding mDia2 full length or DAD tagged with 3×FLAG at the C-terminus. Anti-FLAG immunoprecipitation was performed followed by immunoblotting. (I) Purified GST-mDia2 full-length and truncated mutants (DAD and FH1-FH2) were incubated with His-mDia1 full-length or GBD-DID. Proteins retained on agarose beads after GST pull-down were blotted with the indicated antibodies. The purified GST or GST fusion proteins were examined by Coomassie blue staining. (J) 293T cells were transiently co-transfected with indicated constructs for 48 hours. The cells were then fixed and subjected to immunofluorescent staining with anti-HA (Red) and anti-FLAG (Green) antibodies. Scale bar, 10μm. The asterisks indicate the specific target protein bands. Error bars represent the SEM of the mean. * *p*<0.05, ** *p*<0.01. Two-tailed unpaired student’s t-test was used to generate the *p* values.

mDia formin proteins control their own activities through an inter-molecular interaction. Specifically, the DID motif binds to the DAD domain, leading to the blockage of the function of formin-homology (FH) domains for linear actin initiation and elongation [31, 32]. Given the highly conserved structure and similar roles of mDia1 and mDia2 in HSPC regeneration, we asked whether they could form hetero-oligomers to regulate individual function. To this end, we first performed a coimmunoprecipitation assay and found that exogenously expressed FLAG-tagged murine mDia2 co-precipitated with ectopically expressed HA-tagged murine mDia1, and vice versa (Fig 4A). To test whether exogenously expressed mDia proteins bind to their endogenous counterparts, we ectopically introduced HA-mDia1 or mDia2-3xFLAG into 293T cells, and then performed immunoprecipitation with anti-HA or anti-FLAG magnetic beads. Indeed, the interaction between HA-mDia1 and endogenous mDia2 was readily detected (Fig 4B, left panel), which was also the same for mDia2-3xFLAG with endogenous mDia1 (Fig 4B, right panel). Furthermore, we showed that immunoprecipitation using a mDia2 antibody also pulled down endogenous mDia1 and vice versa (Fig 4C), confirming the interaction of mDia1 and mDia2 *in vivo*.

To identify the specific domain on mDia2 that binds to mDia1, we constructed vectors expressing various truncated mDia2 mutants (Fig 4D). Data from immunoprecipitation assays demonstrate that the C-terminal DAD domain mediates the interaction with mDia1 (Fig 4E). Notably, the DAD domain (amino acids 1033–1171) pulled down much more amount of mDia1 than the mDia2 full-length protein, suggesting increased mDia2 DAD binding to mDia1 in the absence of autoinhibition. Using the same approach, we mapped the region of mDia1 required for mDia2 binding, which revealed that the GBD-DID domain (amino acids 1–567), particularly the DID domain, mediates the interaction of mDia1 with mDia2 (Fig 4F and 4G). mDia1 mutant lacking DAD domain(ΔDAD) also partially bound to mDia2, suggesting DAD of mDia1 may also contribute to the interaction. However, mDia1 DAD was not detectable by immunoblotting largely due to its size (76 amino acids), which hampered our effort to evaluate its role in protein interaction. We further ectopically expressed mDia1 GBD-DID, mDia2, or mDia2 DAD and performed the immunoprecipitation assay. mDia1 GBD-DID was confirmed to interact with mDia2 full-length protein (Fig 4H). Consistently, the mDia2 DAD again displayed more significant interaction with mDia1 GBD-DID, suggesting mDia1 GBD-DID and mDia2 DAD are required and sufficient for their interaction (Fig 4H). To determine whether the interaction of mDia1 and mDia2 is direct, we generated and purified bacterial-expressed recombinant full-length proteins and truncated mutants. Purified GST-mDia2 full-length and DAD were able to interact with His-mDia1 full-length and GBD-DID under cell-free conditions, suggesting a direct interaction between mDia1 and mDia2 (Fig 4I). We next examined the subcellular localization of the mDia1-mDia2 complex and transfected vectors expressing HA-mDia1 and FLAG-mDia2 into 293T cells. Immunofluorescence staining revealed that mDia1 colocalized with mDia2 predominantly in the cytoplasm, with a lesser extent in the nucleus (Fig 4J). To examine whether the mDia1-mDia2 interaction depends on the activities of formin proteins, we treat the cells either with the formin agonist IMM-01, the formin inhibitor SMIFH2, or challenged the cells with serum starvation. The immunoprecipitation results suggest that the formin activity is likely to be dispensable for the mDia1-mDia2 complex formation as either the activation or inhibition treatment at varied concentrations minimally affected the interaction (S6A Fig). However, we indeed observed a slightly enhanced endogenous interaction between mDia1 and mDia2 when cells suffered serum deprivation (S6B Fig), indicating either Rho GTPase or other growth factor-related signaling could potentially modulate the hetero-oligomers formation. Additionally, we observed a significant interaction of mDia1 and mDai2 in the K562 cell, a human erythroleukemic cell line (S6C Fig). Taken together, these data indicate that mDia1 and mDia2 form hetero-oligomers mainly through the interaction between the mDia1 GBD-DID domain and mDia2 DAD domain.

### mDia1 associates with mDia2 to maintain HSPC functions and hematopoiesis

**Fig 5:**
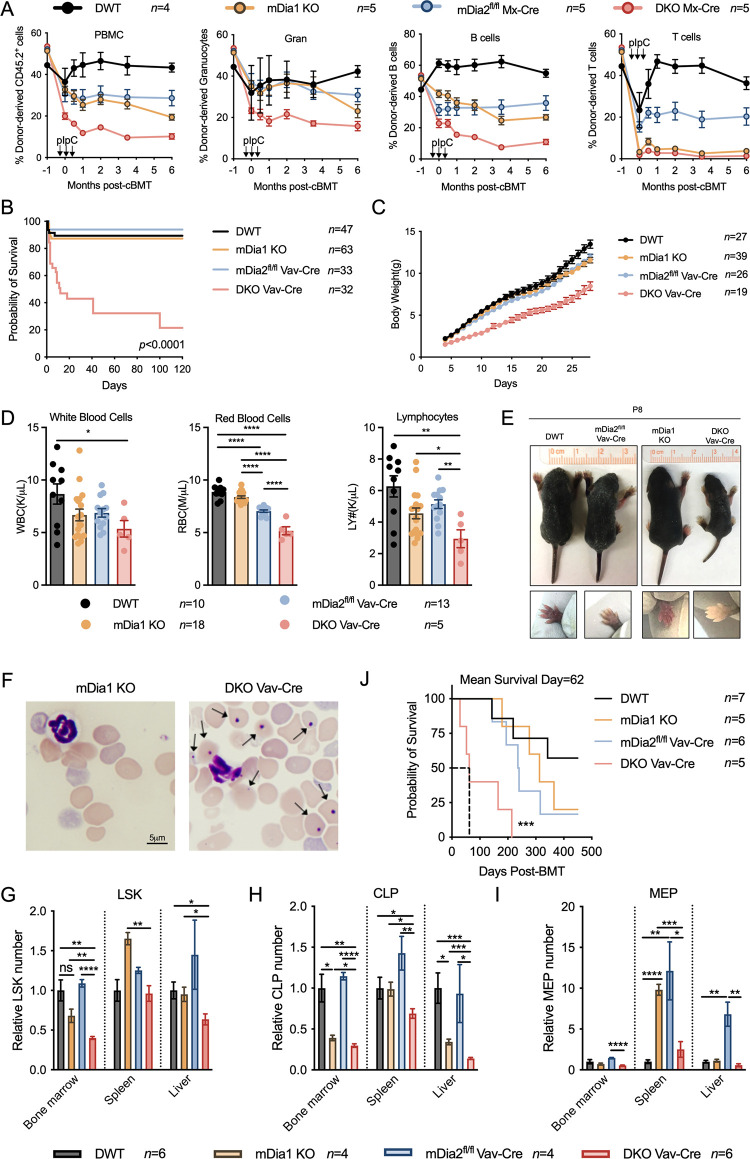
mDia1 cooperates with mDia2 to regulate HSPC functions and hematopoiesis under a steady state and during stress. (A) Chimerism study of the peripheral blood from CD45.1+ recipient mice transplanted with BMMCs (2x10^6^) from either double wild type (DWT), mDia1^-/-^ (mDia1 KO), mDia2^fl/fl^ Mx-Cre or mDia1^-/-^/mDia2^fl/fl^ Mx-Cre (DKO Mx-Cre) mice before pIpC treatment. An equal number of BMMCs from wild-type CD45.1+ mice were used as competitors. The mice were treated with pIpC one month after transplantation. (B) Kaplan-Meier survival curve of the indicated mice. (C) Growth curve of the survived mice in B. (D) Reduction of white blood cell, red blood cell, and lymphocyte counts in DKO Vav-Cre mice. Peripheral blood was obtained from one-month-old indicated mice. DKO Vav-Cre offspring were pooled from 5–10 breeding cohorts. (E) Representative pictures showing reduced body size (upper) and severe anemia as indicated by pale forelimb in DKO Vav-Cre newborns on P8 (lower). (F) Wright-Giemsa stains of the peripheral blood smear from neonates in D. (G-I) Flow cytometric analysis and quantification of HSPCs (LSK) (G), common lymphoid progenitors (CLP) (H), and megakaryocytes-erythroid progenitors (MEP) (I) in infant mice. Data are presented as relative numbers to wild-type control mice. (J) Kaplan-Meier survival curve of indicated recipient mice receiving 5x10^5^ donor BMMCs from neonatal mice in G. Error bars represent the SEM of the mean. * *p*<0.05, ** *p*<0.01, *** *p*<0.001 and **** *p*<0.0001. All the *p* values were generated by a two-tailed unpaired student’s *t-test*, except the *p-value* in J was analyzed by the Mantel-Cox log-rank test.

We next determined the physiologic roles of mDia formins *in vivo* by using mDia1 and mDia2 double knockout mice. We crossed mDia1 whole-body knockout and mDia2^fl/fl^ Mx-Cre mice and generated inducible and hematopoietic specific mDia1/2 double knockout mice (DKO Mx-Cre). Following injection of synthetic double-stranded RNA (dsRNA) analog, polyinosinic:polycytidylic acid (polyIC), the *Cre* recombinase expression is activated and driven by the Mx promoter in an interferon-dependent manner. We performed a competitive bone marrow transplantation assay using an equal amount of BMMCs from these mice and CD45.1+ wild-type competitors. Once the engraftment was established (one month after transplantation), we treated the recipient mice with polyIC followed by chimerism analyses to determine the role of mDia formins in HSPC maintenance. As expected, donor cells with loss of either mDia1 or mDia2 were significantly outcompeted over time by their WT counterparts, which was more prominent with dual deficiency of mDia1 and mDia2 (Fig 5A). Notably, DKO Mx-Cre donor cells exhibited compromised reconstitution even before polyIC treatment in the initiating timepoint, which may be attributed to the spontaneous Mx-Cre expression and subsequent deletion of the floxed genes in hematopoietic cells upon transplantation [33]. These results indicate that mDia1 could associate with mDia2 to regulate HSPC integrity. HSPCs with mDia1 and mDia2 dual deficiency lose their capacity over time to maintain in the niche under a steady state.

To further examine the roles of mDia formins in HSPC maintenance, we crossed mDia1 KO and mDia2^fl/fl^ Vav-Cre mice and generated *mDia1*^-/-^ *mDia2*^*fl/fl*^ Vav-Cre (DKO Vav-Cre) mice. Consistent with our previous report [25], survival of mDia2^fl/fl^ Vav-Cre mice was comparable to wild-type mice although they showed anemia. mDia1 KO mice develop MDS when they are over one year old but do not have compromised survival at younger ages [22]. In stark contrast, loss of both mDia1 and mDia2 in the late stage of embryonic development (Vav is expressed at approximately embryonic day 13) caused increased lethality postnatally and during the first 6 months of life (Figs 5B and S7A). Mice that survived postnatally also showed a failure to thrive and pancytopenia compared to their single knockout counterparts (Fig 5C–5E). Due to the difficulties in obtaining sufficient adult DKO Vav-Cre mice, we dissected mice on postnatal day 8 (P8). Splenomegaly was observed in both DKO Vav-Cre and single knockout neonatal mice, in which DKO Vav-Cre mice showed the highest ratio of the spleen to body weight (S7B Fig). DKO Vav-Cre neonates also displayed small body sizes with paler forelimbs (Fig 5E). Morphologic examination of peripheral blood smear revealed dramatically increased Howell-Jolly bodies due to severe anemia (Figs 5F and S7C). Furthermore, flow cytometric analysis revealed profoundly decreased LSK (Fig 5G) and CLP (Fig 5H) populations in the bone marrow of DKO Vav-Cre mice, although its bone marrow MEP (Fig 5I), CMP (S7D Fig), and GMP (S7E Fig) did not alternate significantly. Extramedullary hematopoiesis, especially the MEP expansion in the spleen and liver in single knockout mice, was also markedly diminished in DKO Vav-Cre mice (Fig 5I). We next performed a bone marrow transplantation of BMMCs from DKO Vav-Cre P8 postnatal mice into lethally irradiated wild-type mice. Compared to the recipients transplanted with BMMCs from single knockout and WT donors, mice transplanted with cells from DKO Vav-Cre exhibited rapid lethality with a mean survival of 62 days (Fig 5J). These data further demonstrate the non-redundant roles of mDia1 and mDia2 in governing HSPC homeostasis under steady state and during transplantation.

### Transcriptional alterations in mDia-deficient HSPCs involve SRF signaling

**Fig 6:**
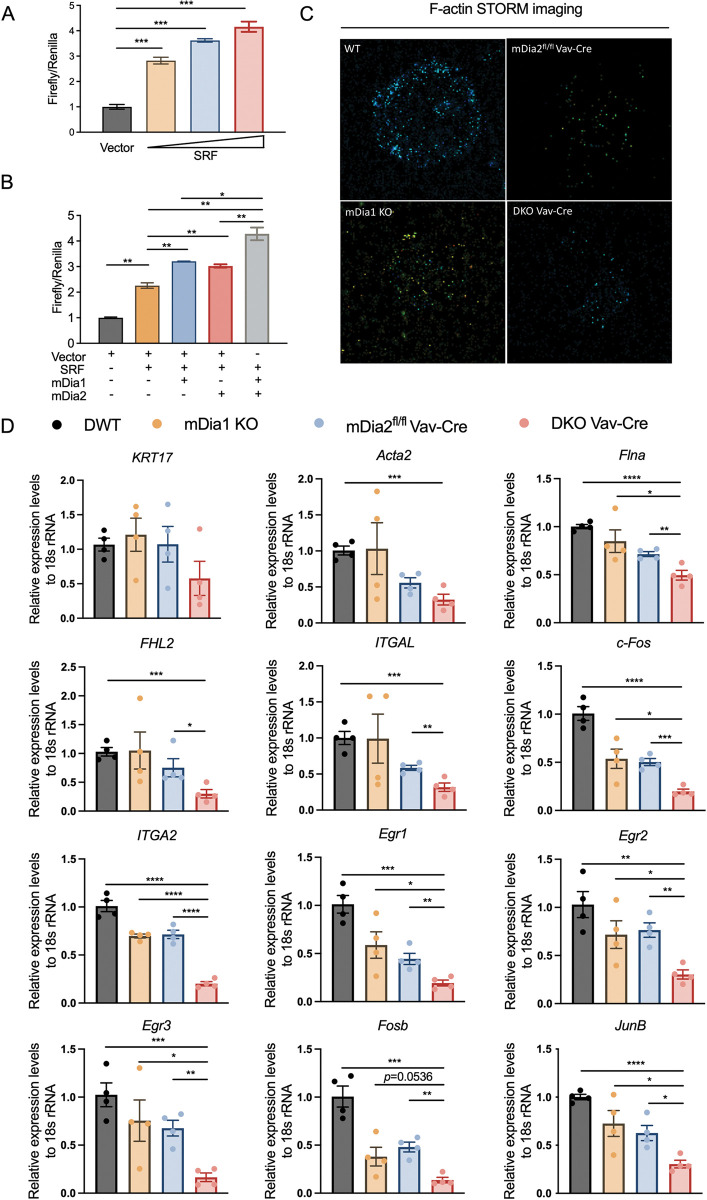
Cooperative regulation of SRF transcriptional activity by mDia1 and mDia2. (A) Dose-dependent response of luciferase activity in SRE-luciferase reporter construct induced by SRF. 293T cells were transfected with reporter plasmid in the presence of vector alone or increasing amount of SRF. The luciferase activity was examined by a dual-luciferase reporter assay system 24 hours post-transfection, and data were pooled from two independent experiments. (B) mDia1 modulates SRF transcriptional activity synergistically with mDia2. Luciferase activity was performed in 293T cells transfected with indicated constructs as in A. Data were pooled from two independent experiments. (C) Bone marrow lineage-negative cells obtained from the indicated mice at P8 were fixed and stained with fluorescent phalloidin for F-actin. Representative images of single-molecule super-resolution STORM imaging were presented. (D) Quantitative PCR analysis of indicated SRF target genes in c-Kit+ HSPCs. 18S rRNA was used as an internal control. Error bars represent the SEM of the mean. * *p*<0.05, ** *p*<0.01, *** *p*<0.001, **** *p*<0.0001. All the *p* values were generated by a two-tailed unpaired student’s *t-test*.

mDia fromins regulate the cytoskeleton remodeling by influencing the gene expression via megakaryocytic acute leukemia (MAL) protein (also known as MRTF-A or MKL1)/serum response factor (SRF) pathway [3, 34]. Specifically, mDia formin-mediated actin polymerization reduces G-actin monomers bound to MAL, leading to the rapid translocation of released MAL into the nucleus. Subsequent dimerization of the co-factor MAL with SRF promotes the expression of SRF target genes. The MAL/SRF activity has been early recognized to be pivotal for seeding and maintaining HSPCs properties by regulating the expression of genes involved in cell tracking, cell adhesion, and chemotactic responses [35, 36]. To determine whether mDia1 and mDia2 co-operatively enhance the transcriptional activity of MAL/SRF, we constructed a luciferase vector containing the conserved CArG box sequences (CC (A/T)_6_GG), also named as SRF responsive element (SRE), obtained from SRF target genes [37–39]. Indeed, exogenously overexpressed SRF triggered dose-dependent induction of SRE-luciferase activity (Fig 6A). As expected, SRF-induced luciferase expression was augmented by mDia1 and mDia2 alone, which was further significantly boosted by co-transfecting them together (Fig 6B). Using stochastic optical reconstruction microscopy (STORM) imaging, we found that F-actin levels significantly declined in HSPCs with either mDia1 or mDia2 depletion, while DKO Vav-Cre HSPCs exhibited the most compromised synthesis of actin filaments (Fig 6C). These data suggest that dual deficiency of mDia formins is likely to result in a downregulation of the SRF transcriptional signature that is critical for HSPCs integrity and maintenance. To test this, we purified c-kit^+^ HSPCs and determined the SRF transcriptional activity by detecting its downstream target gene expression through a quantitative real-time PCR assay. Indeed, the canonical SRF target genes, including *Flna*, *FHL2*, *ITGAL*, *c-Fos*, *ITGA2*, *Egr1*, *Egr2*, *Egr3*, *Fosb*, and *JunB* exhibited compromised transcription at different levels upon either mDia1 or mDia2 depletion (Fig 6D). Combined deficiency of mDia1 and mDia2 further significantly inhibited these aforementioned gene expressions in HSPCs. Hence, mDia1 and mDia2 could coordinate the SRF transcriptional activity in HSPCs, which may account for the defective murine hematopoiesis in DKO mice.

## Discussion

We recently revealed the critical roles of mDia1 in the pathogenesis of MDS and subsequent leukemic transformation [18, 22, 28, 40], however, its role in HSPC integrity has not been previously explored. Given the important functions of mDia2 in HSPC lodgment in the niche [3], we reasoned that mDia1 may also be involved in the HSPC function. In this study, we demonstrate that mDia1 is critical in HSPCs stemness and fitness as shown by declined regeneration and engraftment abilities of mDia1 deficient cells, which could contribute to the pathogenesis of MDS with loss of mDia1. Additionally, the human gene encoding mDia1 is located between the proximal and distal common deleted region (CDR) on chromosome 5q. Previously, we have shown that mDia1 is downregulated in the bone marrow of del(5q) and 5q- MDS patients, which is associated with aberrant overexpression of CD14 that contributes to the development of MDS through sensitized innate immune responses [22]. It is unclear whether the expression of mDia2 in myeloid neoplasms is altered. It will be interesting to examine whether mDia1 insufficiency in human MDS could compromise the mDia2 activity, leading to the collapse of the cellular cytoskeleton.

HSPCs lose their self-renewal capacity and reconstitution potential upon transplantation during aging. We previously revealed that aging mDia2 deficient HSPCs exhibit compromised engraftment during the transplantation [3], which is similar to the defect observed in mDia1 null HSPCs in this study. When further analyzing the HSPCs in the old mDia2 conditional knockout mice, we found that old mDia2 deficient mice do not exhibit pancytopenia as observed in mDia1 KO mice. In addition, there was a mild HSPC expansion during aging with loss of mDia2, in contrast to the declined HSCPs in old mDia1 deficient mice. Furthermore, when cell quiescence was examined, we did not detect any differences between control and mDia2 knockout mice. These data demonstrate that mDia1 and mDia2 contribute differently to maintaining HSPCs homeostasis during aging.

Precise cell cycle regulation is critical for HSPCs since the loss of cell quiescence and entry into the cycling often lead to stem cell exhaustion. In fact, mDia formin proteins have been implicated in cell cycle regulation. For instance, mDia1 was shown to regulate the cell cycle of mouse myoblasts via SRF-dependent and SRF-independent transcriptional control of MyoD expression [41]. Lack of mDia1 and mDia3 expression led to the reduced separation of the centrosome from the nucleus [26]. mDia1 was further found to be localized to the mitotic spindle [42], and depletion of mDia1 caused mitosis failure in Hela cells [43]. In addition, ubiquitylation of mDia2 is largely dependent on the cell cycle [44]. Dysregulation of its expression or activity has been demonstrated to cause cytokinesis failure in tumor cells [44], fibroblasts [45], and fetal and adult erythroblasts [23, 25]. Specifically, By modulating the incorporation of the centromere-specific histone H3 variant, CENP-A, and nuclear actin polymerization, mDia2 governs the centromere movement [46, 47]. mDia2 further ensures the accurate segregation of chromosomes in neural progenitors via interaction with the key regulator of the spindle assembly checkpoint, BubR1 [48]. We found that, after transplantation, mDia1 KO LSK cells exhibited decreased G0 and increased cycling cells in S-G2/M. while the transplanted mDia2 KO LSK cells tend to have fewer cells in the G0 phase but increased in the G1 phase. The consistently declined G0 cells in both mDia1 and mDia2 LSK cells in our current study further demonstrate that both of them are required for maintaining hematopoietic stem cell quiescence or cell cycle under transplantation stress, yet the underlying mechanisms remain elucidative. RNA transcriptome assay, particularly for single-cell RNA sequencing, of HSPCs from both single and dual-deficient mice may offer more clues.

Erythropoiesis and HSPCs maintenance or hierarchy are influenced by different cytokines, especially for erythropoietin (EPO) and thrombopoietin (TPO) [49]. EPO is produced mainly in the kidney, liver, and brain [50, 51], whereas TPO is primarily generated in the liver [52]. In our current study, although mDia1 null mice is a constitutively knockout strain, mDia2 is specifically deleted in hematopoietic lineages. Therefore, we expect limited effects of mDia2 in other tissues. Moreover, mDia1 deficient mice do not exhibit anemia throughout their life (Fig 2A and [22]). Despite mDia2 deficient mice showing anemia due to binucleated erythroblast formation, its phenotype is erythroid cell autonomous with no evidence of microenvironment involved [25]. Most importantly, both mDia1 and mDia2 deficient LSK displayed compromised cell cycle only under bone marrow transplantation settings (Fig 1E and [3]), in which the EPO and TPO are mainly generated from wild-type kidney, liver, and brains from receipt mice. Therefore, we expect that there should be minimal effects of EPO or TPO in HSPCs in our single knock-out models. Nonetheless, altered cellular cytoskeletons can indeed lead to cytokine dysregulation, engaging in host defense and cell-autonomous immunity [22, 28, 53]. Upon simultaneous depletion of mDia1 and mDia2, we observed more severe anemia and declined HSPCs in double mutant mice. In this way, we cannot exclude the possibility that compound loss of mDia1 and mDia2 might affect cytokines such as EPO and (or) TPO production *in vivo*, which needs further exploration.

Non-redundant functions of mDia proteins have been previously recognized in cortical microtubule capturing and cell migration [54]. Recently they were also implicated in controlling primary ciliogenesis [55, 56]. Moreover, mDia1 and mDia3 regulate tangential migration of cortical and olfactory inhibitory interneurons in mouse brains [26]. They also generate cortical F-actin meshwork in sertoli cells to ensure murine spermatogenesis and male fertility [57]. T cell receptor (TCR) activation of immune synapse requires mDia1/3-dependent polymerization and subsequent ring formation of F-actin in mice as well [14]. Using the hematopoietic-specific mDia2 knockout and conventional mDia1 null mice, we examined the contributions of each formin protein in maintaining HSPC integrity and stemness. Our results demonstrate that mDia1 and mDia2 are both critical for HSPCs functions, as evidenced by decreased cell numbers during transplantation and aging stress, as well as reduced colony formation in serial plating or delayed recovery when chemotherapy challenges were applied. We further defined the non-redundant and collaborative roles of mDia1 and mDia2 in murine hematopoiesis. Specifically, double-deficient mice had increased mortality due to anemia and cytopenia with declined HSPCs. F-actin synthesis was significantly compromised upon double knockout, which led to the suppression of SRF transcriptional signaling. Our work provides insights into dissecting associations among mDia homologs and a paradigm for their non-redundant roles under different conditions.

In contrast to the lack of anemia in mDia1 knockout mice, mDia2 conditional knockout and DKO models exhibit anemia, although all the mutant mice have splenomegaly. The splenomegaly in mDia1 KO mice is largely due to myeloid neoplasms as we and others reported [22, 28, 29]. Furthermore, we previously showed that binucleated late-stage erythroblast due to cytokinesis failure causes anemia in mDia2 deficient mice [25]. Accordingly, the ineffective erythropoiesis in the bone marrow of mDia2 knockout mice triggers the extramedullary erythropoiesis in the spleen [25]. The erythroid defects in mDia2 deficient cells are worsened by the loss of mDia1 expression. HSPC defects in DKO mice could be the major underlying reason, yet the specific mechanisms remain to be further studied. More efforts need to be taken to investigate erythroid cell production, maturation, and hemolysis or phagocytosis of RBCs in the DKO mouse model.

Compromised filament-actin synthesis caused by actin cytoskeleton dysregulation usually triggers the cellular G-actin accumulation, which in turn attenuates SRF transcriptional activity by sequestering the co-activator MAL in the cytosol[3, 34, 58, 59]. We observed additive inhibition of F-actin formation by STORM-imaging in double mutant cells. We further validated the suppressed SRF signaling cascades by quantitatively verifying the reduced expression levels of SRF canonical targets in c-kit^+^ DKO HSPCs. However, given that enforced expression of canonical SRF target FHL2, one critical regulator of stem cell quiescence[60], was unable to restore the engraftment defect as observed in mDia1 KO cells (S8A-S8C Fig), some limitations in our experiments should be noted. Firstly, SRF-independent pathways may also play roles in our mouse models, as similarly observed in mouse myoblasts [41]. Secondly, the altered cell distribution may influence gene expression in c-Kit+ HSPCs. Nonetheless, since the alteration trend of CMP/GMP/MEP in single and double KO mice does not match the gene expression profiling, our quantitative PCR results may still reflect the compromised SRF transcriptional activity in double mutant HSPCs. Future unbiased single-cell analyses could overcome these limitations.

Previous work suggests that mDia proteins tend to assemble as homodimers to regulate actin cytoskeleton and microtubule dynamics [9, 61]. Accumulating evidence also indicates that interactions between different formin proteins are possible. For example, inverted formin 2 (INF2) was shown to be able to bind mDia proteins [62]. Inhibition of Rho signaling by co-expressed C3 transferase was previously reported to be required for the exogenous mDia1 and mDia2 protein association [63]. In the current work, we provide direct evidence that mDia1 and mDia2 can exist as hetero-oligomers *in vitro* and *in vivo* regardless of the Rho signaling or other protein factors. Interestingly, previous proteomic analyses also identified mDia2 precipitated with mDia3 or mDia1 [54, 64]. Therefore, it is likely that formin monomer protein might be exchangeable during oligomerization, which warrants further investigations.

**Fig 7:**
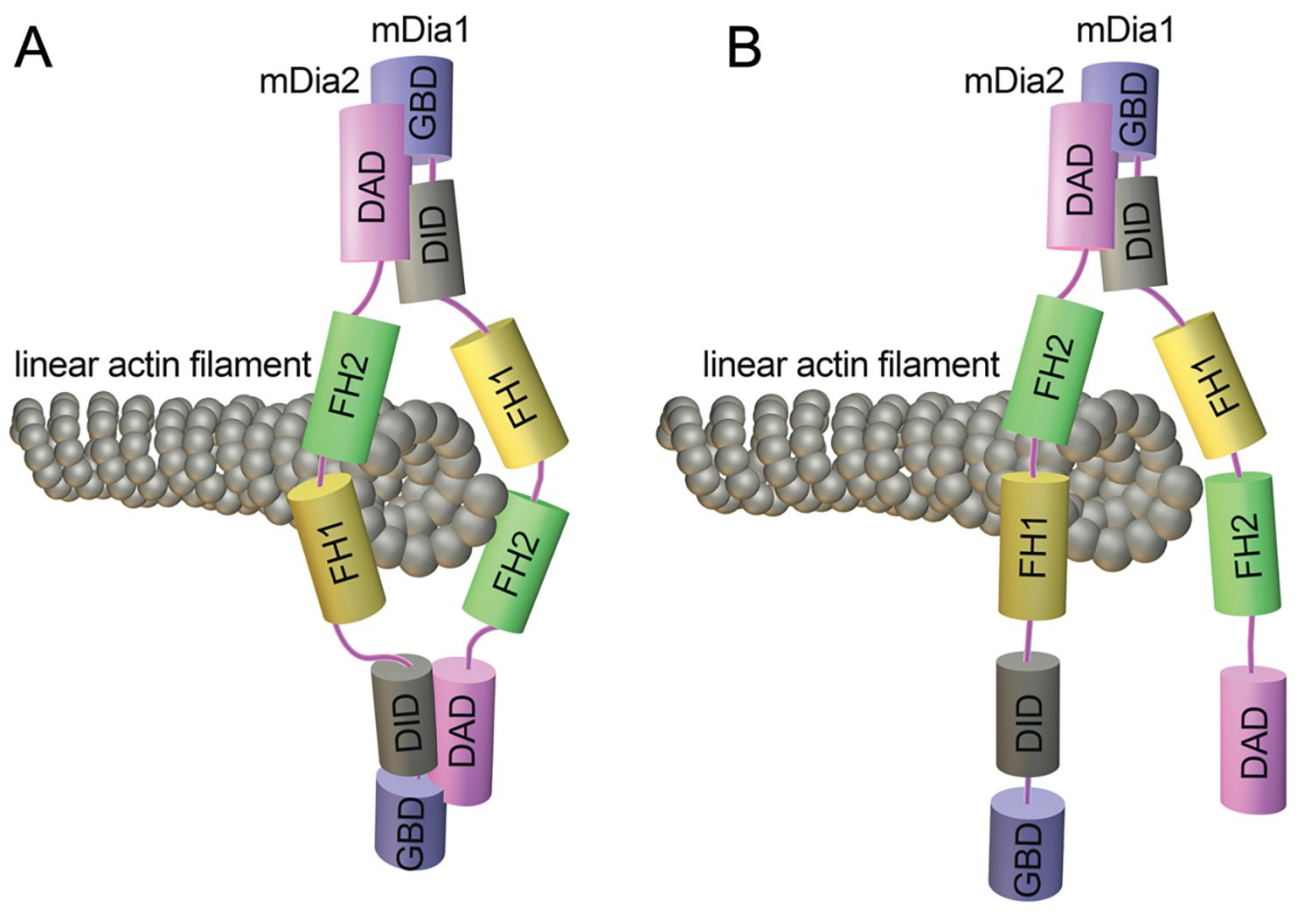
Schematic of mDia heterodimers in hematopoiesis. A. A putative bracelets-like structure composited by mDia1-mDia2 interaction. (B) A newly proposed active zipper-like confirmation assembled by mDia1-mDia2 heterodimer in this study.

We uncovered that mDia1 GBD-DID domains were critical for the interaction with mDia2 DAD. Similar to our results, the INF2-DID domain interacts with mDia-DAD [62]. Notably, we found that mDia2 GBD-DID or DID alone is insufficient to bind to mDia1. Similarly, mDia1 DAD is not involved in binding to mDia2. It appears that similar domains from different formins harbor diverse properties or binding affinities, and the potential heterodimers tend to be in a zipper-like conformation instead of a bracelets-like structure [9, 61, 63] (Figs 7, S9A and S9B). It should be noted that INF2 binding to mDia proteins antagonized the SRF-responsive gene transcription and Rho-activated actin polymerization in podocytes [62, 65]. In contrast, our work demonstrated that mDia1-mDia2 oligomerization coordinately contributes to actin organization and augments SRF transcriptional activity in HSPCs. Our work has also limitations in that mDia1-mDia2 interaction has not been verified in the context of HSPCs due to unavailable specific antibodies recognizing endogenous murine mDia2 for immunoprecipitation, however, we identified the protein association in the human K562 erythroleukemia cell line. Further studies will be needed to reveal the formin complex formation in more stem-like cells and to define whether and how the hetero- or homo-dimerization is temporally or spatially regulated as well as to what extent it might play physiological roles in different cell types.

## Methods

### Ethics statement

All the experiments involving animals were conducted in accordance with the Guide for the Care and Use of Laboratory Animals and were approved by the Institutional Animal Care and Use Committee (IACUC) at both Northwestern University and Hunan University.

### Mice

The mDia1 whole-body knockout mice and hematopoietic specific mDia2 deficient mice (*mDia2*^*fl/fl*^ *Mx-Cre* and *mDia2*^*fl/fl*^ *Vav-Cre*) have been previously described [3, 22, 25]. The *mDia1*^*-/-*^ *mDia2*^*fl/fl*^ *Vav-Cre* or *mDia1*^*-/-*^ *mDia2*^*fl/fl*^ *Mx-Cre* double mutant mice were obtained by crossing mDia1 knock-out mice with *mDia2*^*fl/fl*^ *Vav-Cre* or *mDia2*^*fl/fl*^ *Mx-Cre* mice, respectively. All the mice were maintained in the background of C57/BL6. The CD45.1-positive congenic mice were purchased from Charles River or Jackson Laboratory.

### Plasmids

The MICD4 (MSCV-IRES-hCD4) construct with truncated human CD4 expression was described previously, and all expression fragments were sub-cloned 5’ prior to the IRES element [24, 66]. MICD4-FLAG-mDia2 was constructed previously [67], and was subcloned into pCDH-ECMV-MCS-EF1α-ZsGreen1-T2A-Puro vector either with full-length mDia2 or truncated mutants fused with triple FLAG tag (3×FALG) at the C-terminus by *OK Clon* DNA Ligation Kit (Catalog #AG11802, Accurate Biology, China) or Ligation high Ver.2 (Catalog #LGK-201, TOYOBO). mDia1 coding sequence with N-terminal FALG was amplified and inserted into MICD4. The full length of HA-mDia1 and variants were subsequently amplified and subcloned into a pLVX-IRES-Neo vector by DNA recombination ligation. The coding sequence of FHL2 was PCR-amplified from mouse bone marrow cDNA and was inserted into MI-tRFP657, a MICD4-based construct in which the human CD4 was replaced by tagRFP657 [68]. For luciferase construct containing the SRF response elements (SRE), quadruple CarG box (CC(A/T)_6_GG)) sequence (CCCTATATGG CCTTATATGG CCATATATGG CCAAATAAGG) were synthesized and cloned into pGL4.22 (Promega, E6771) between *KpnI* and *BglII*. The oligonucleotide sequences for cloning experiments have been shown in S1 Table.

### *In vivo* 5-FU treatment

3-months old sex-matched littermate control and mDia2 conditional knockout mice were administered by i.p. injection of 5-fluorouracil (freshly dissolved in 1×PBS at 7.5 μg/μl) (Catalog #F6627, Sigma) at 150mg/kg of body weight. Approximately 100ul aliquots of peripheral blood collected from both groups were analyzed on day 7 after 5-FU injection for differential cell counts. The mice were further challenged with 5-FU on day 7 and day 14 respectively to monitor the survival rates. The survivals were sacrificed on day 22, and the bone marrow cellularity and HSPCs were assayed by flow cytometer.

### Flow cytometric assays

The flow cytometric analyses of mouse bone marrow, spleen, and hematopoietic stem/progenitor cells were described previously [3, 28].

### Colony-forming unit assay

Upon initial plating, a total of 1×10^4^ murine BMMC cells per well were cultured in 1mL of methylcellulose medium (Methocult M3234, STEMCELL Tech.) containing 50ng/ml SCF, 10ng/ml IL-3, 10ng/ml IL-6 and 3U/ml Erythropoietin (EPO, Catalog # GH002, HumanCells Bio.) and 1×penicillin and streptomycin in 6-wells plate. After incubation at 37°C under 5% CO_2_ and high humidity for 14 days, colony-forming units (CFUs) (>50 cells) were scored according to the manufacturer’s instructions under Olympus CKX31 inverted microscope. For serial re-plating experiments, the cells from colonies were pooled and dissociated into single cells by pipetting with PBS. 1×10^4^ cells per well were further seeded for another 14 days. To qualify colony size, the colonies were photographed by Olympus IX51 with cellSens Entry software. The colony area was calculated by Image J (1.45s).

### Bone marrow transplantation

Non-competitive transplantation (BMT) and competitive transplantation (cBMT) were performed using mouse bone marrow cells as previously described [3, 22, 25, 28].

### *In vitro* HSPCs expansion and infection

Bone marrow c-Kit+ HSPCs were purified using a mouse CD117 (c-Kit)-positive selection kit (STEMCELL Tech.) following the manufacturer’s instructions. The purified c-Kit+ cells were then cultured in a PVA-based serum-free expansion medium [69]. Briefly, 3×10^6^ c-Kit+ HSPCs were seeded within Ham‘s F-12K Medium (Catalog #21127022, Thermo Fisher Scientific), supplemented with 0.1% PVA (Catalog #P8136, Sigma), 1% Insulin-Transferrin-Selenium-Ethanolamine(ITS-X) (100X) (Catalog #51500056, Thermo Fisher Scientific), 10mM HEPES(N-2-hydroxyethylpiperazine-N-2-ethane sulfonic acid) (Catalog #15630130, Thermo Fisher Scientific), 1% Penicillin-Streptomycin-L-Glutamine Solution (100X) (Catalog #10378016, Thermo Fisher Scientific), 100ng/ml mouse TPO (Catalog #50146-M08H, SinoBiological), and 10ng/ml mouse SCF(Catalog #78064, Stemcell Technologies) for 14 days, incubated at 37°C with 5% CO2 incubator. Retroviral particles were generated as previously described [3], except Polyethylenimine (PEI) (Catalog #23966, Polysciences) was used as the transfection reagent. Viral supernatants were collected 48 h after transfection, and debris was removed by brief centrifugation. Retroviral infection of c-Kit+ HSPCs was performed by suspending the cells in freshly prepared viral supernatants in the presence of 10μg/ml polybrene (Catalog #40804ES86, YEASEN) and centrifuged at 900g for 90 min at 37°C. After spin-infection, the viral supernatants were gently removed and the cells were incubated with fresh PVA-medium and cultured for another 36–48 hours *in vitro* followed by subsequent bone marrow transplantation.

### Homing assay

The homing assay was performed as described before [3]. Briefly, BMMCs (CD45.2+, 2×10^6^) or bone marrow lineage-negative cells (CD45.2+, 1×10^7^) from wild-type or mDia1 KO mice were mixed with an equal number of competitive BMMCs or lineage-negative cells (CD45.1+). The cell mixtures were retro-orbitally injected into lethally irradiated (X-Ray, Rad Source RS-2000, 7.5Gy) wild-type CD45.1+/CD45.2+ recipient mice. BMMCs or lineage-negative cells were harvested from the tibia and femur 16 hours post-injection for flow cytometric analysis. The cell percentages of CD45.1+ versus CD45.2+ were determined.

### Complete blood cell counts

Peripheral blood was collected by retro-orbital (RO) bleeding and stored in tubes containing K2EDTA. Complete blood cell counts were examined by Hemavet 950 instrument (Drew Scientific) or XN-1000 automatic hematology analyzer (Sysmex).

### Western blotting

The cells were lysed by the IP lysis buffer (50mM HEPES, pH7.6, 250mM NaCl, 5mM EDTA, pH8.0, 0.1% NP40) containing protease inhibitor cocktail (Catalog #M5293, AbMole bioscience) on ice for 30 minutes followed by once freezing/thawing cycle at -80°C. The concentration of the protein lysate was determined by the BCA protein Assay kit (Catalog #E-BC-K318-M, Elabsciences). An equal amount of protein from all the samples was prepared with the sample buffer and was further separated on the SDS-PAGE gel after boiling for 10 minutes. Subsequently, the proteins were transferred to a 0.45μm PVDF membrane (Catalog #IPVH00010, Millipore). After blocking with 5% milk for 1 hour at room temperature, the membrane was incubated with appropriate primary antibodies at 4°C overnight. After washing, the blots were incubated with HRP-conjugated secondary antibodies (Abiowell) for 1 hour at room temperature and the HRP signals were detected by the ECL Chemiluminescence Detection Kit (Catalog #P1020, Applygen). The following antibodies were used: anti-FLAG (Catalog #20543-1-AP, Proteintech), anti-HA (Catalog #51064-2-AP, Proteintech), anti-mDia1 (Catalog #20624-1-AP, Proteintech), anti-mDia1 (Catalog #610848, BD Pharmingen), anti-mDia2 (Catalog #14342-1-AP, Proteintech), anti-His (Catalog #66005-1-Ig, Proteintech), and anti-GAPDH (Catalog #AP0066, Bioworld).

### Immunoprecipitation

The cell lysate preparation and protein concentration determination were described in Western blotting. For immunoprecipitation with endogenous protein, cell lysate containing 1 -1.5mg total protein was applied for incubation with 2.5μg anti-DIAPH1, anti-DIAPH3, or IgG (Catalog #sc-2025, Santa Cruz) at 4°C for overnight followed by protein A/G magnetic beads incubation at room temperature for 2 hours. For immunoprecipitation of ectopically overexpressed protein, 500μg total protein lysate was incubated with anti-FLAG magnetic beads (Catalog #L-1011, Biolinkedin) directly at 4°C overnight. The immuno-complex was washed 6 times with IP buffer and subsequently subjected to Western blotting.

### GST pull-down

*E*. *coli* BL21-DE3 were induced to express GST, GST-mDia2-FL, GST-mDia2-DAD, and GST-mDia2-FH1-FH2 fusion proteins for 16 hours at 20°C with 10μM, 20μM, 40μM and 50μM IPTG (Catalog #B541007, Sangon Biotech) respectively. The bacterial pellets were subsequently lysed by NETN buffer (0.5% NP40, 100uM EDTA, 20mM Tris, 300mM NaCl) containing PMSF (Catalog #A100754, Sangon Biotech) with sonication followed by binding to the glutathione agarose (Catalog #16100, Thermo Fisher) at 4°C for 2 hours. Protein-bound glutathione agarose beads were recovered by NETN buffer washing and were then incubated with the lysate from *E*. *coli* BL21-DE3 expressing His-mDia1-FL or His-mDia1-GBD-DID (1μM IPTG, 20°C, 16 hours) at 4°C for 2 hours. The beads were extensively washed 5 times by the NETN100 buffer (100μM EDTA, 20mM Tris, 100mM NaCl) and subjected to Western blotting.

### Dual luciferase activity assays

The dual luciferase activity assay was conducted as we previously performed[3]. Briefly, HEK293T cells (1.25×10^5^ cells per well) were split into 48-well plates and incubated for 18–24 hours (around 70–80% confluence prior to transfection). Co-transfections were performed by using Neofect DNA transfection reagent with pGL4.22-SRE (100ng/well) together with MICD4 empty vector (300ng/well) or with an increased amount of murine SRF expressing construct (MICD4-mSRF, 50ng/well, 150ng/well and 300ng/well) in Fig 6A, MICD4-mSRF (50 ng/well) with or without mDia1 and (or) mDia2 expressing constructs (MICD4-mDia1 or MICD4-mDia2, 125ng/well) in Fig 6B. *Renilla* luciferase expression vector pRL-TK (1 ng per well) was co-transfected for internal control. The cells were harvested 24 hours post-transfection, and luciferase activity was measured using the Dual-Luciferase Reporter Assay System (E1910, Promega) according to the manufacturer’s instructions. *Firefly* luciferase activities were normalized to *Renilla* luciferase activities (Firefly/Renilla) and calculated as the fold-change to an empty vector. All the luciferase experiments were performed in triplicate.

### Cell quiescence analysis

The stem cell quiescence was profiled by pyronin Y staining as previously described [3].

### Quantitative real-time RT-PCR

The RNA isolation, complementary DNA synthesis, and quantitative real-time PCR were performed as in previous investigations [3, 22, 25, 28]. The primer sequences have been summarized in S2 Table.

### Stochastic optical reconstruction microscopy (STORM) imaging

The STORM imaging analysis was performed as described previously [70, 71].

### Molecular modeling by Alphafold2 predictions

Predicted structures were calculated by AlphaFold Multimer (2.3.1) [72] running on GPU nodes of the High-Performance Computing Center of Central South University. Each job was run on a single node consisting of 4 x Tesla V100 NVlink 32 GB GPUs. The best model (determined by pTM score) was collected from multiple predictions generated from each run. Molecular graphics and analyses were performed with UCSF ChimeraX [73].

### Statistics

All results are presented as mean ± SEM. Statistical comparisons between the two groups were performed by a two-tailed unpaired Student’s t-test using GraphPad Prism software (version 8.0). Survival curves were compiled using Kaplan-Meier algorithms in Prism software, and the significance was assessed using the Mantel-Cox log-rank test. *p* value less than 0.05 was considered statistically significant.

## Supporting information

S1 Fig mDia1 is largely dispensable for HSPCs at the steady stage. (A) Representative flow cytometric plots showing the gating strategies for HSPCs. (B-C) The hematopoietic progenitor cells from 3 months-old WT and mDia1 KO mice were assayed by flow cytometric analysis. Percentages and absolute cell count of indicated cell populations were shown in B and C respectively. n.s., not significant.
(TIFF)

S2 Fig mDia1 is not required for HSPCs homing to bone marrow. (A-C) WT or mDia1 KO BMMCs (A) or lineage-negative (CD45.2+) HSPCs (B-C) were mixed with equal wild-type CD45.1+ competitive cells and transplanted into lethally irradiated receipt mice (CD45.1+/CD45.2+). Bone marrow chimerism was determined 16 hours after transplantation. *n* = 4 per group for A, and *n* = 3 per group for B-C.
(TIFF)

S3 Fig mDia1 transcription is not altered in HSPCs during aging. (A) UCSC browser track from aging HSC Epigenome [30] showing the DNA methylation (red), H3K36me3 peaks (dark blue), H3K4me3 for transcription start sites (TSS, pink), and RNA expression (green, RNA-Seq) of actively transcribed regions of the *Diap1* gene locus in 4 months- and 24 months-old HSCs. (B) *Diap1* transcription levels from A were further quantified and shown.
(TIFF)

S4 Fig mDia regulates HSPCs aging and colony formation under re-seeding stress. (A) Peripheral blood cell counts of 2-year-aged control and mDia2 conditional KO mice (mDia2^fl/fl^ Vav-Cre). (B) Flow cytometric analyses of the percentage and total cell counts of indicated HSPC subpopulations in the bone marrow from mice in A. (C) Proportion of bone marrow LSK cells in each stage of the cell cycle (G0, G1, S-G2/M) from the indicated aged mice. (D-E) Quantification of CFU numbers as in D and colony size as in E with serial plating of bone marrow cells from indicated wild type or mDia1 KO mice performed in triplicate. 1°: 125 colonies in WT, and 133 colonies in mDia1 KO; 2°: 286 colonies in WT, and 225 colonies in mDia1 KO. Error bars represent the SEM of the mean. *p < 0.05, **p < 0.01, ***p < 0.001, ****p < 0.0001. Two-tailed unpaired student’s t-test was used to generate the *p* values.
(TIFF)

S5 Fig mDia formins are required for cell recovery in 5-FU-induced myeloid suppression. (A) The numbers of HSPCs and committed progenitors from purified lineage-negative cells were analyzed and quantified by flow cytometer analysis by day 7 after 5-FU treatment. (B) Survival percentage of lineage-negative cells from A assayed by Annexin V staining. (C) Complete blood cell counts of wild-type or mDia1 KO mice were determined by day 7 after the first injection of 5-FU. (D) Kaplan-Meier survival analysis of indicated mice challenged with serial 5-FU injection. Error bars represent the SEM of the mean. * *p*<0.05, ** *p*<0.01, *** *p*<0.001, **** *p*<0.0001. Two-tailed unpaired student’s t-test was used to generate the *p* values.
(TIFF)

S6 Fig mDia1 interacts with mDia2 independent of formin activity. (A) Co-immunoprecipitation assays were performed in 293T cells with mDia2-3×FLAG overexpression treated with IMM-01 or SIMFH2 at the indicated dose for 24 hours, followed by Western blotting of the indicated proteins. (B) 293T cells either untreated (Control) or treated with serum starvation (SS) for 6hr or SS followed by adding 20% serum back (6hr) (SS+20%FBS) were collected for immunoprecipitation assay. Western blotting analyses with indicated antibodies following the anti-mDia1 IP were shown. (C) Co-Immunoprecipitation assay using an anti-mDia1 antibody was performed in K562 cells followed by Western blotting with indicated antibodies.
(TIFF)

S7 Fig mDia1 and mDia2 double deficiency significantly influence HSPC functions *in vivo*. (A) The percentages of mDia1/mDia2 double knockout mice expressing Vav-Cre (DKO Vav-Cre) in the end of weaning time, day 21(P21), or immediately after birth (P1) in the indicated breeding strategies screened by genotyping PCR. (B) Images showing splenomegaly in mDia1 or mDia2 single deficient and DKO mice (left). Quantitative analyses of the spleen versus body weight (right). (C-D) Quantification of CMP and GMP cell numbers in the indicated neonates by flow cytometry analysis at P8 was shown in C and D respectively. Data are presented as relative numbers to double wild-type control mice. *n* = 6 in DWT, *n* = 4 in mDia1 KO, *n* = 4 in mDia2^fl/fl^ Vav-Cre, *n* = 6 in DKO Vav-Cre. Error bars represent the SEM of the mean. *p < 0.05, **p < 0.01, ***p < 0.001, ****p < 0.0001. Two-tailed unpaired student’s t-test was used to generate the *p* values.
(TIFF)

S8 Fig Enforced expression of FHL2 is insufficient for restoring the engraftment defects in mDia1 KO HSPCs. (A-C) The c-Kit+ HSPCs from wild-type or mDia1 KO mice were transduced with retroviruses expressing either empty vector or murine FHL2 followed by competitive transplantation, in which the CD45.1+ c-kit+ HSPCs infected with empty vector served as the competitors. The relative mRNA expression levels of *FHL2* were determined 48 hours after viral transduction by quantitative PCR (A). The infection efficiency (B) and engraftment (C) were determined by flow cytometric analysis of peripheral blood chimerism from transplants one month post-transplantation. *n* = 5 in each group for B-C. Error bars represent the SEM of the mean. *p < 0.05, **p < 0.01, ***p < 0.001, ****p < 0.0001. Two-tailed unpaired student’s t-test was used to generate the *p* values. n.s., not significant.
(TIFF)

S9 Fig Molecular modeling of mDia1 and mDia2 hetero-oligomerization structure. (A-B) Protein structural prediction by Alphalfold 2 illustrates that mDia1 GBD-DID is proximately located with mDia2 DAD (A), while mDia2 GBD-DID prefers to be isolated from mDia1 DAD (B).
(TIFF)

S1 Table Primer sequences for molecular cloning. (DOCX)

S2 Table Primer sequences for real-time quantitative PCR. (DOCX)

S1 Source Data Numerical data underlying figures. (XLSX)
