# Supplementary figures and images for: mDia formins form hetero-oligomers and cooperatively maintain murine hematopoiesis
Source: PLoS Genet. 2023 Dec 29;19(12):e1011084. doi: 10.1371/journal.pgen.1011084 (PMC10756686; doi:10.1371/journal.pgen.1011084)

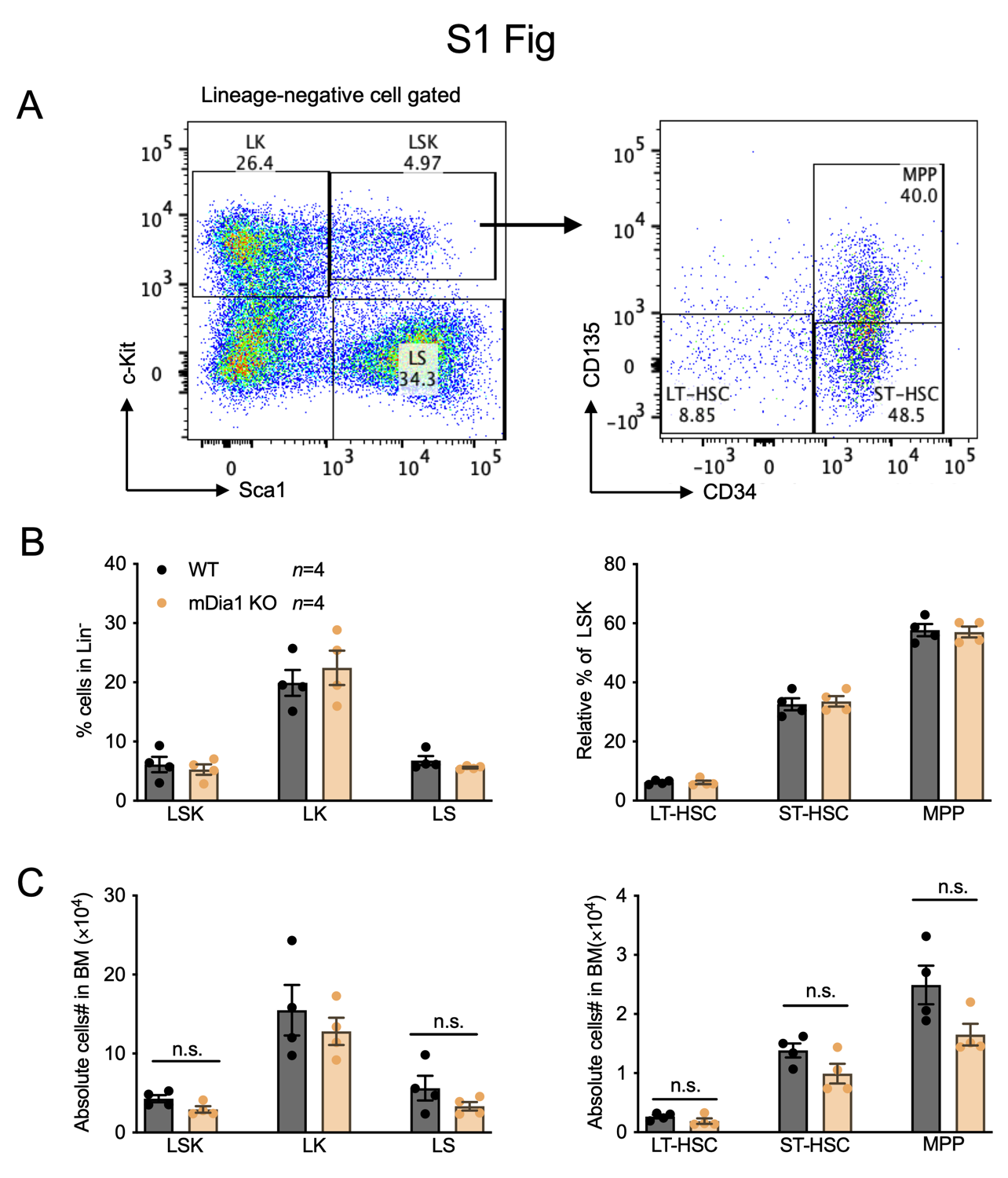

Supplement: S1 Fig — (A) Representative flow cytometric plots showing the gating strategies for HSPCs. (B-C) The hematopoietic progenitor cells from 3 months-old WT and mDia1 KO mice were assayed by flow cytometric analysis. Percentages and absolute cell count of indicated cell populations were shown in B and C respectively. n.s., not significant. (TIFF) [file pgen.1011084.s001.tiff]

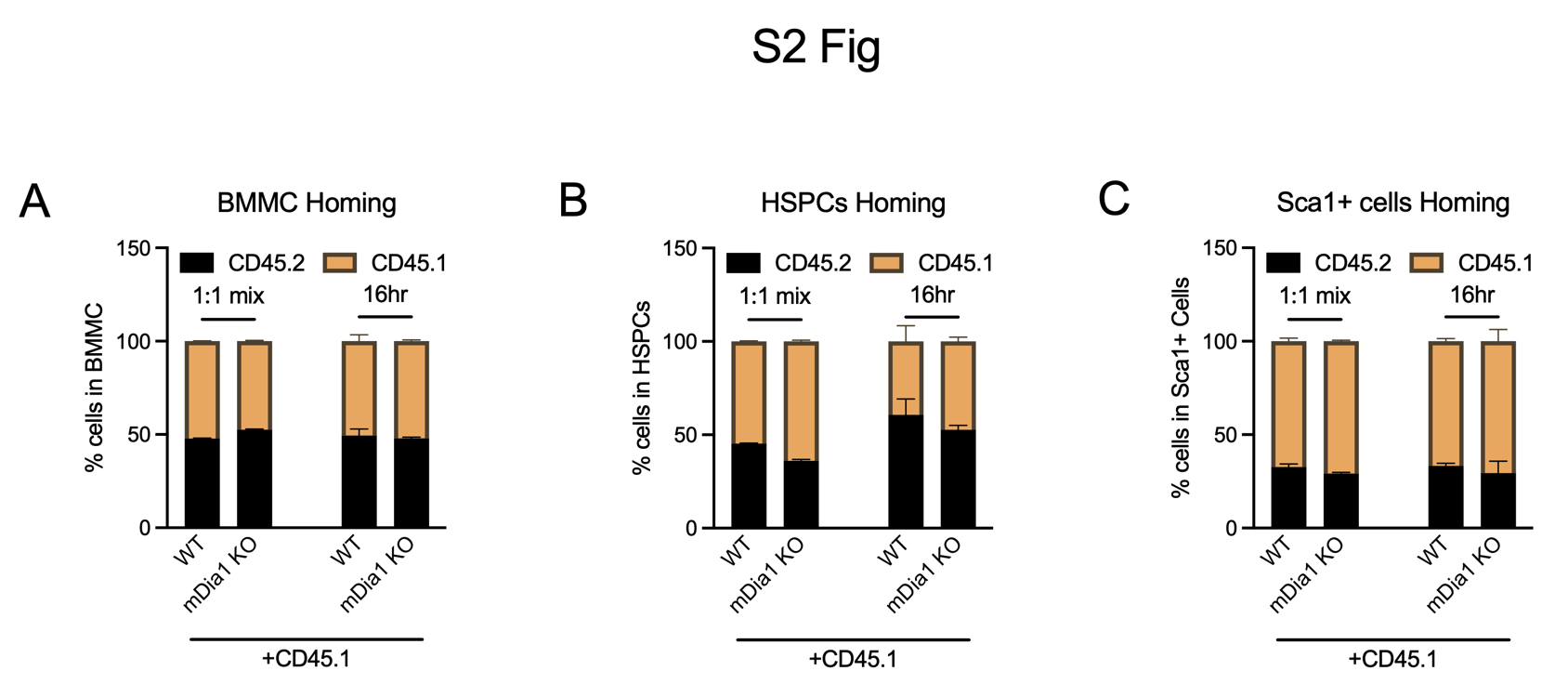

Supplement: S2 Fig — (A-C) WT or mDia1 KO BMMCs (A) or lineage-negative (CD45.2+) HSPCs (B-C) were mixed with equal wild-type CD45.1+ competitive cells and transplanted into lethally irradiated receipt mice (CD45.1+/CD45.2+). Bone marrow chimerism was determined 16 hours after transplantation. n = 4 per group for A, and n = 3 per group for B-C. (TIFF) [file pgen.1011084.s002.tiff]

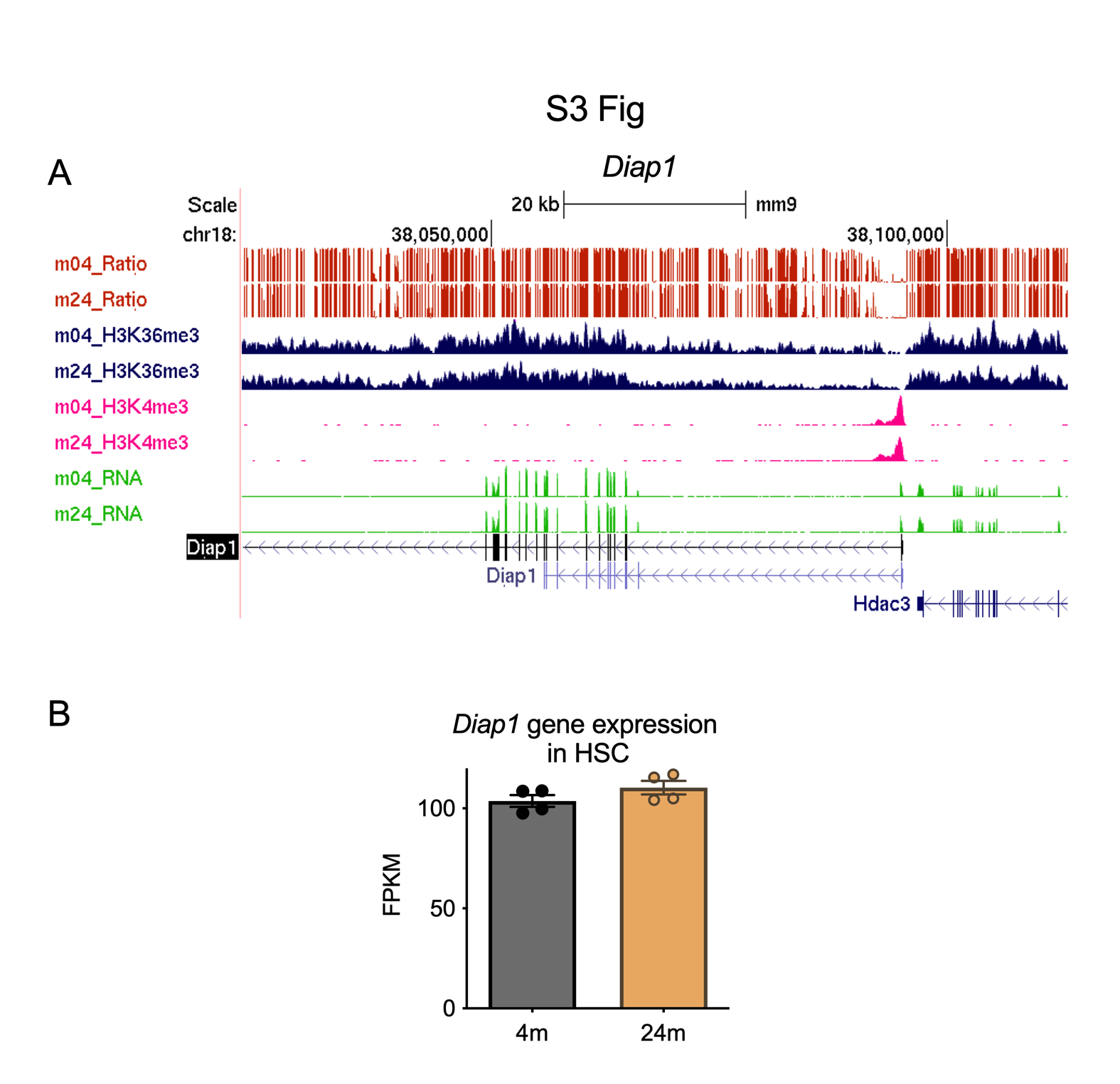

Supplement: S3 Fig — (A) UCSC browser track from aging HSC Epigenome [30] showing the DNA methylation (red), H3K36me3 peaks (dark blue), H3K4me3 for transcription start sites (TSS, pink), and RNA expression (green, RNA-Seq) of actively transcribed regions of the Diap1 gene locus in 4 months- and 24 months-old HSCs. (B) Diap1 transcription levels from A were further quantified and shown. (TIFF) [file pgen.1011084.s003.tiff]

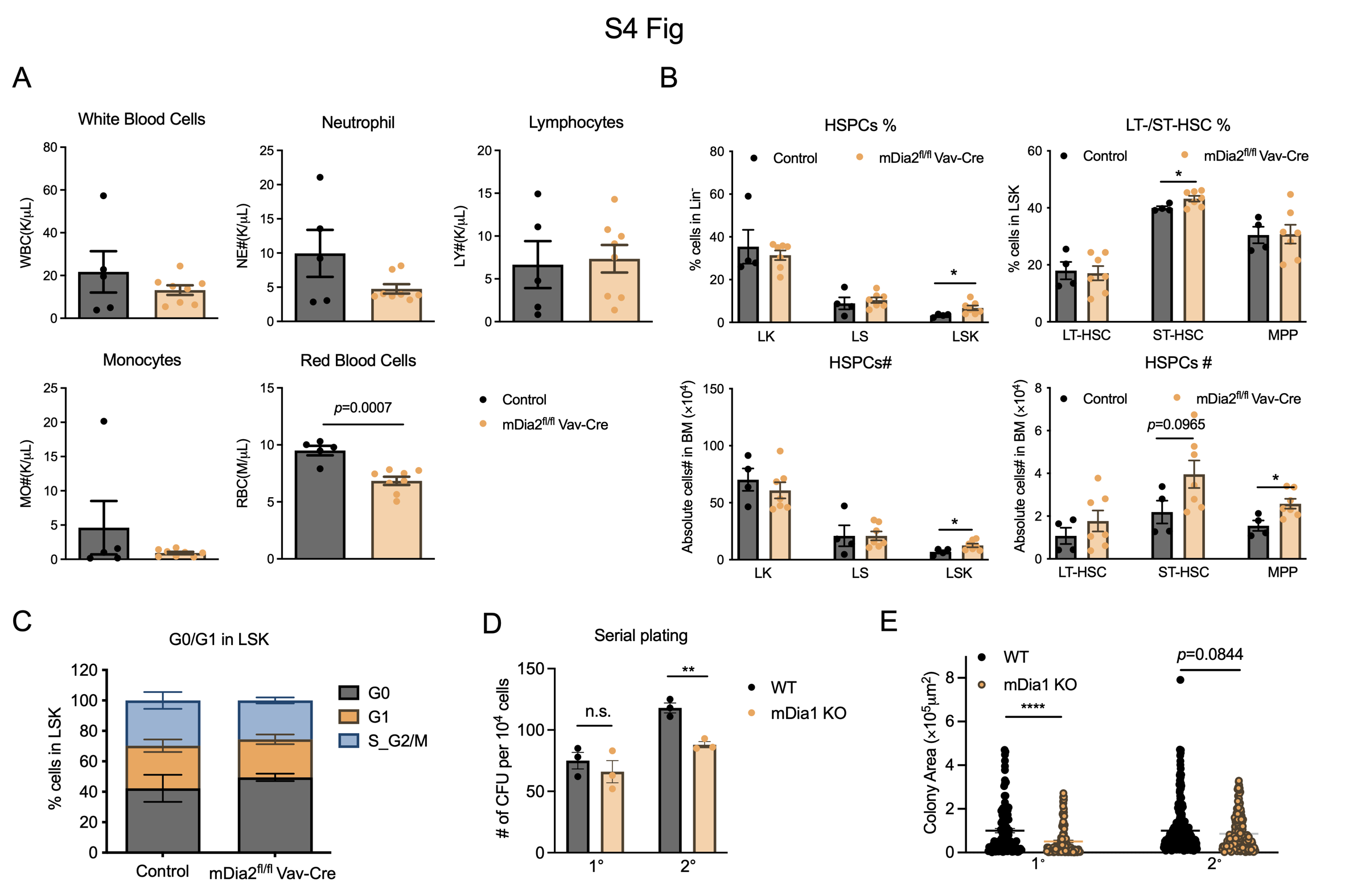

Supplement: S4 Fig — (A) Peripheral blood cell counts of 2-year-aged control and mDia2 conditional KO mice (mDia2fl/fl Vav-Cre). (B) Flow cytometric analyses of the percentage and total cell counts of indicated HSPC subpopulations in the bone marrow from mice in A. (C) Proportion of bone marrow LSK cells in each stage of the cell cycle (G0, G1, S-G2/M) from the indicated aged mice. (D-E) Quantification of CFU numbers as in D and colony size as in E with serial plating of bone marrow cells from indicated wild type or mDia1 KO mice performed in triplicate. 1°: 125 colonies in WT, and 133 colonies in mDia1 KO; 2°: 286 colonies in WT, and 225 colonies in mDia1 KO. Error bars represent the SEM of the mean. *p < 0.05, **p < 0.01, ***p < 0.001, ****p < 0.0001. Two-tailed unpaired student’s t-test was used to generate the p values. (TIFF) [file pgen.1011084.s004.tiff]

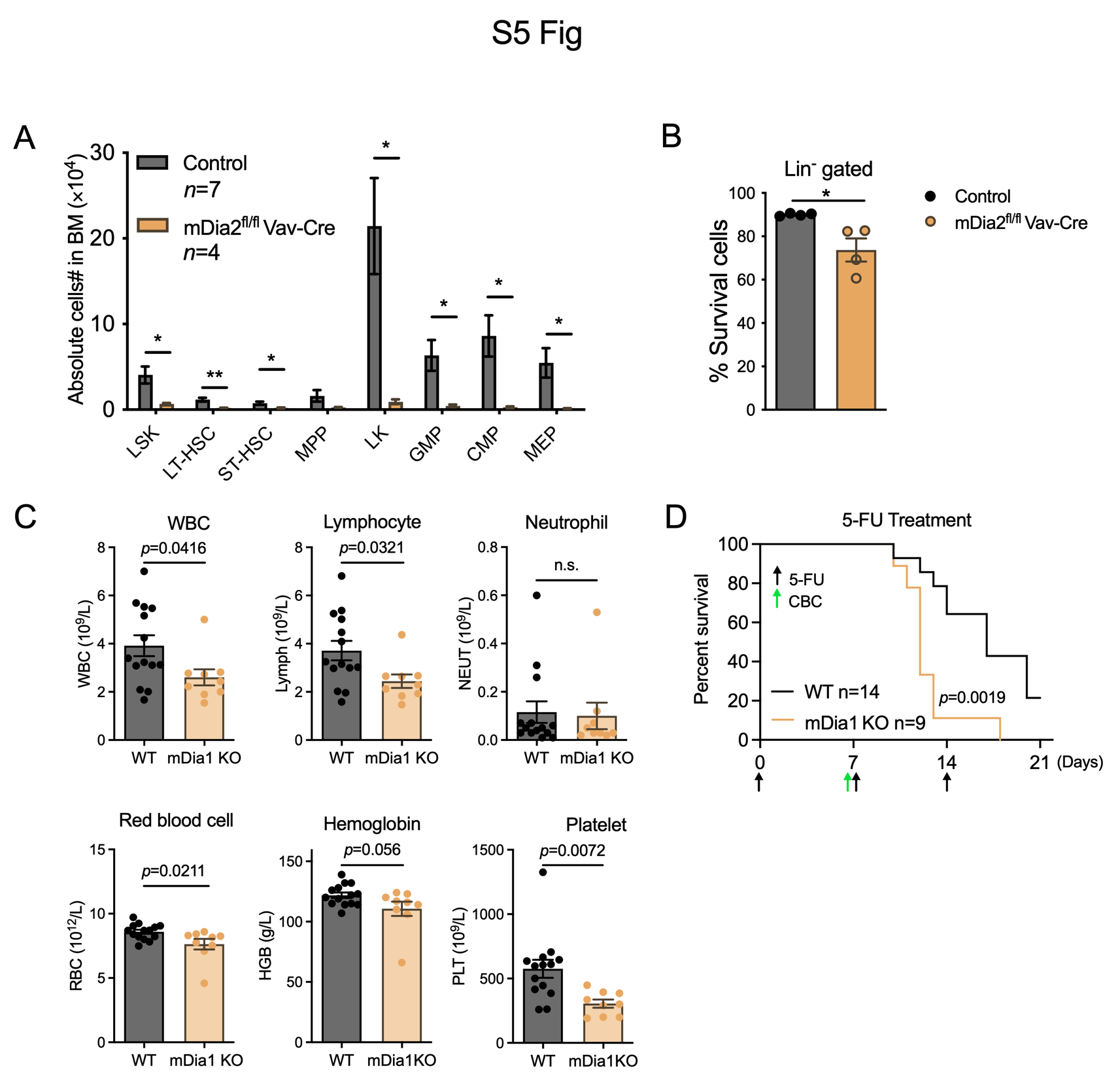

Supplement: S5 Fig — (A) The numbers of HSPCs and committed progenitors from purified lineage-negative cells were analyzed and quantified by flow cytometer analysis by day 7 after 5-FU treatment. (B) Survival percentage of lineage-negative cells from A assayed by Annexin V staining. (C) Complete blood cell counts of wild-type or mDia1 KO mice were determined by day 7 after the first injection of 5-FU. (D) Kaplan-Meier survival analysis of indicated mice challenged with serial 5-FU injection. Error bars represent the SEM of the mean. * p<0.05, ** p<0.01, *** p<0.001, **** p<0.0001. Two-tailed unpaired student’s t-test was used to generate the p values. (TIFF) [file pgen.1011084.s005.tiff]

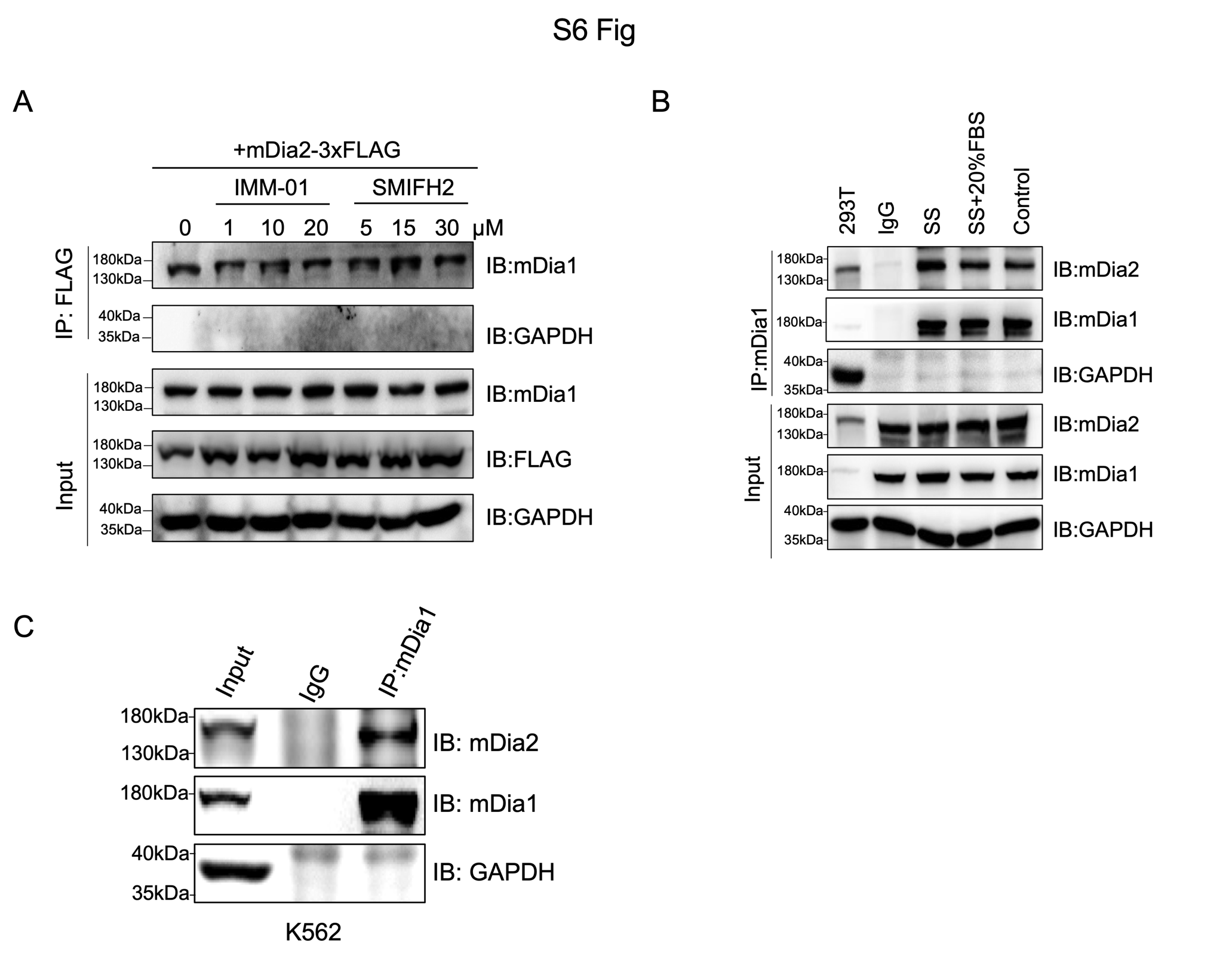

Supplement: S6 Fig — (A) Co-immunoprecipitation assays were performed in 293T cells with mDia2-3×FLAG overexpression treated with IMM-01 or SIMFH2 at the indicated dose for 24 hours, followed by Western blotting of the indicated proteins. (B) 293T cells either untreated (Control) or treated with serum starvation (SS) for 6hr or SS followed by adding 20% serum back (6hr) (SS+20%FBS) were collected for immunoprecipitation assay. Western blotting analyses with indicated antibodies following the anti-mDia1 IP were shown. (C) Co-Immunoprecipitation assay using an anti-mDia1 antibody was performed in K562 cells followed by Western blotting with indicated antibodies. (TIFF) [file pgen.1011084.s006.tiff]

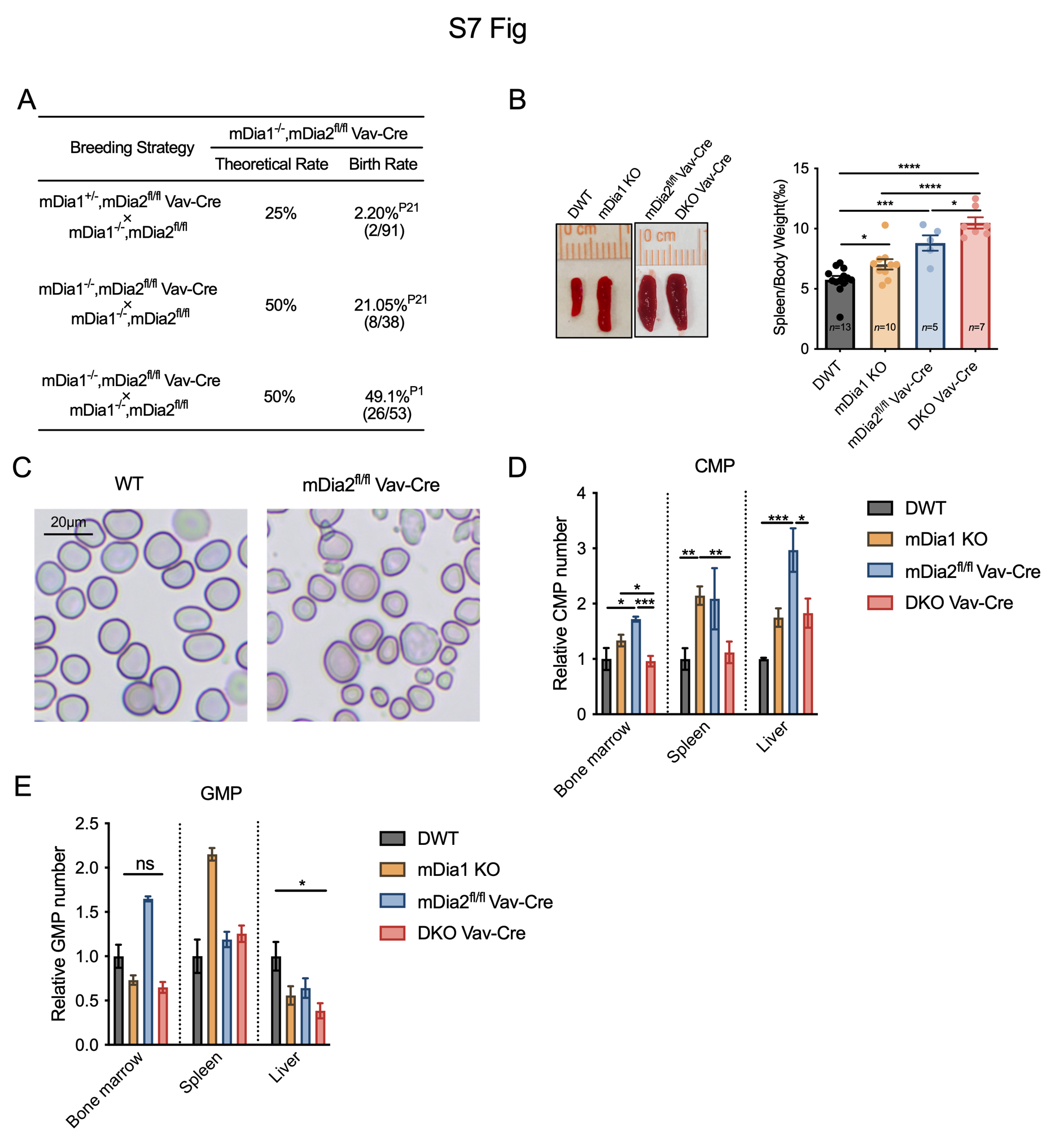

Supplement: S7 Fig — (A) The percentages of mDia1/mDia2 double knockout mice expressing Vav-Cre (DKO Vav-Cre) in the end of weaning time, day 21(P21), or immediately after birth (P1) in the indicated breeding strategies screened by genotyping PCR. (B) Images showing splenomegaly in mDia1 or mDia2 single deficient and DKO mice (left). Quantitative analyses of the spleen versus body weight (right). (C-D) Quantification of CMP and GMP cell numbers in the indicated neonates by flow cytometry analysis at P8 was shown in C and D respectively. Data are presented as relative numbers to double wild-type control mice. n = 6 in DWT, n = 4 in mDia1 KO, n = 4 in mDia2fl/fl Vav-Cre, n = 6 in DKO Vav-Cre. Error bars represent the SEM of the mean. *p < 0.05, **p < 0.01, ***p < 0.001, ****p < 0.0001. Two-tailed unpaired student’s t-test was used to generate the p values. (TIFF) [file pgen.1011084.s007.tiff]

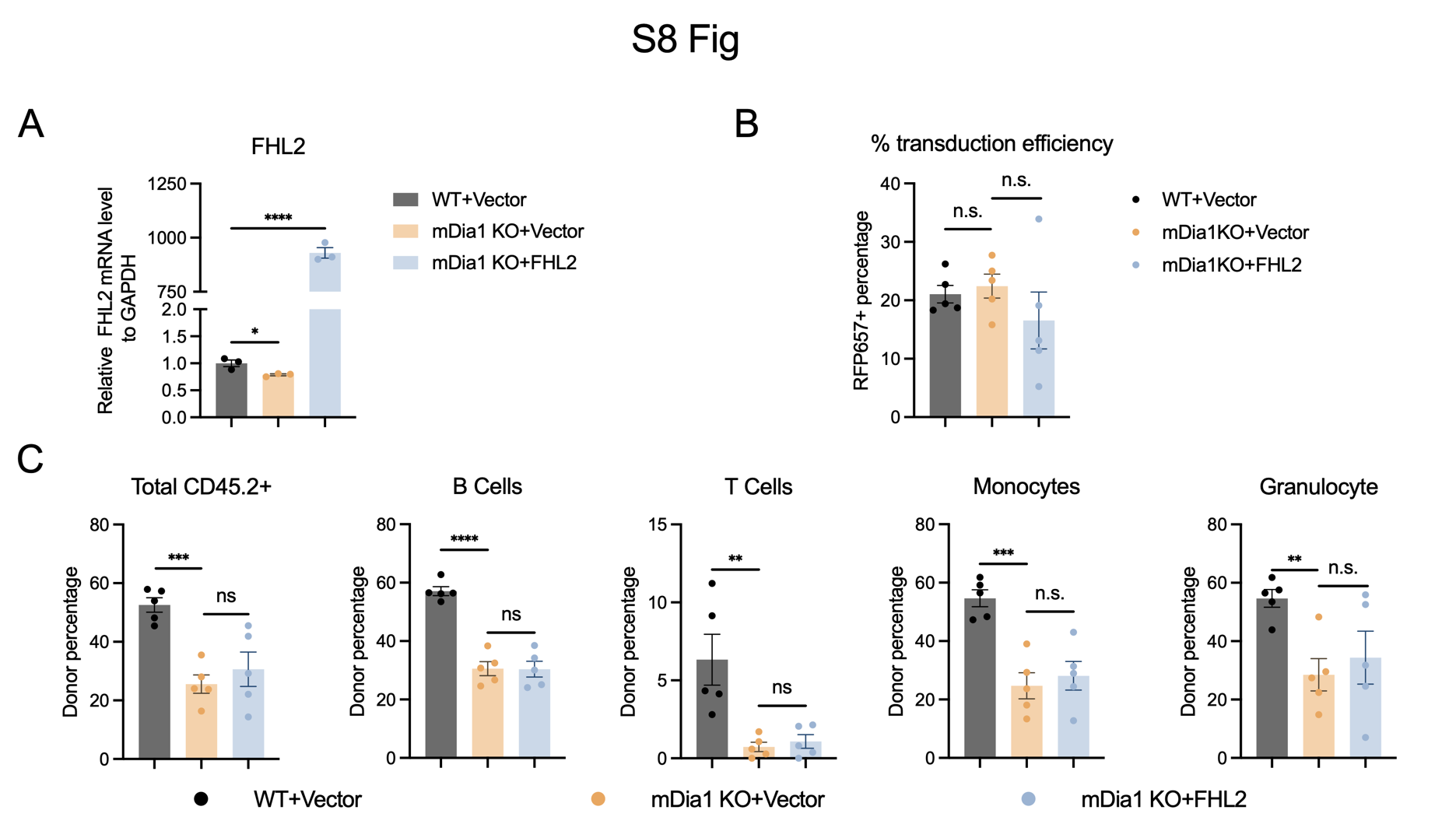

Supplement: S8 Fig — (A-C) The c-Kit+ HSPCs from wild-type or mDia1 KO mice were transduced with retroviruses expressing either empty vector or murine FHL2 followed by competitive transplantation, in which the CD45.1+ c-kit+ HSPCs infected with empty vector served as the competitors. The relative mRNA expression levels of FHL2 were determined 48 hours after viral transduction by quantitative PCR (A). The infection efficiency (B) and engraftment (C) were determined by flow cytometric analysis of peripheral blood chimerism from transplants one month post-transplantation. n = 5 in each group for B-C. Error bars represent the SEM of the mean. *p < 0.05, **p < 0.01, ***p < 0.001, ****p < 0.0001. Two-tailed unpaired student’s t-test was used to generate the p values. n.s., not significant. (TIFF) [file pgen.1011084.s008.tiff]

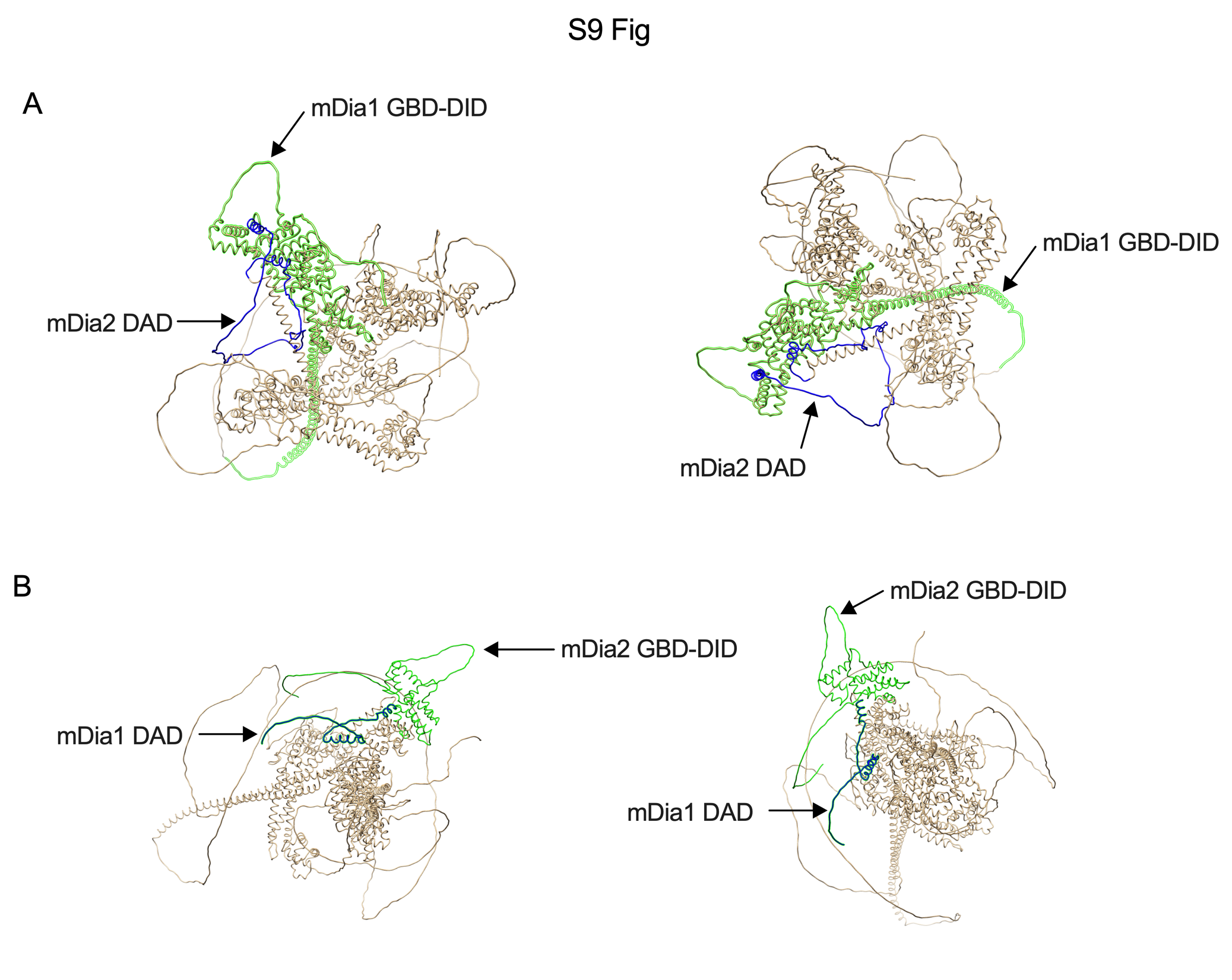

Supplement: S9 Fig — (A-B) Protein structural prediction by Alphalfold 2 illustrates that mDia1 GBD-DID is proximately located with mDia2 DAD (A), while mDia2 GBD-DID prefers to be isolated from mDia1 DAD (B). (TIFF) [file pgen.1011084.s009.tiff]
