# Supplementary material for: mDia formins form hetero-oligomers and cooperatively maintain murine hematopoiesis
Source: PLoS Genet. 2023 Dec 29;19(12):e1011084. doi: 10.1371/journal.pgen.1011084 (PMC10756686; doi:10.1371/journal.pgen.1011084)
Supplement: S1 Table — (DOCX) [file pgen.1011084.s010.docx]

**S1 Table.** Primer sequences for molecular cloning.

| **Primer Name** | **Sequence (5’ > 3’)** | **Notes** |
| --- | --- | --- |
| mDia2 FL | | |
| mDia2-FL-F | ggatctatttccggtgaattcGCCACCATGGAGAGGCACC | Forward and reverse primers for PCR amplification of mDia2 full length with homology arms complementary to pCDH vector. |
| mDia2-DAD-R | taagcttggtaccgaggatccTAAAGCTCGTAATCTTGCCAGCA |  |
| mDia2-GBD-DID | | |
| mDia2-FL-F | ggatctatttccggtgaattcGCCACCATGGAGAGGCACC | Forward and reverse primers for PCR amplification of mDia2-GBD-DID with homology arms complementary to pCDH vector. |
| mDia2-GBD-DID-R | taagcttggtaccgaggatccGGCACCAAACTGGGATTTAAAA |  |
| mDia2-GBD | | |
| mDia2-FL-F | ggatctatttccggtgaattcGCCACCATGGAGAGGCACC | Forward and reverse primers for PCR amplification of mDia2-GBD with homology arms complementary to pCDH vector. |
| mDia2-GBD-R | taagcttggtaccgaggatccGGCTTTTGCCAACAAGGAAA |  |
| mDia2-DID | | |
| mDia2-DID-F | ggatctatttccggtgaattcGCCACCATGGATCCCAGG | Forward and reverse primers for PCR amplification of mDia2-DID with homology arms complementary to pCDH vector. |
| mDia2-DID-R | taagcttggtaccgaggatccGGCACCAAACTGGGATTTAAAA |  |
| mDia2-FH1-FH2 | | |
| mDia2-FH1-FH2-F | ggatctatttccggtgaattcGCCACCATGTTGCCACCTGGTACAAA | Forward and reverse primers for PCR amplification of mDia2-FH1-FH2 with homology arms complementary to pCDH vector. |
| mDia2-FH1-FH2-R | taagcttggtaccgaggatccAGTTTTCATTTCTAGTAAGCGCTTTTT |  |
| mDia2-FH1 | | |
| mDia2-FH1-FH2-F | ggatctatttccggtgaattcGCCACCATGTTGCCACCTGGTACAAA | Forward and reverse primers for PCR amplification of mDia2-FH1 with homology arms complementary to pCDH vector. |
| mDia2-FH1-R | taagcttggtaccgaggatccCAGAGGAGGTGGTGGTGGTACA |  |
| mDia2-FH2 | | |
| mDia2-FH2-F | ggatctatttccggtgaattcGCCACCATGGGATTCCTGGGT | Forward and reverse primers for PCR amplification of mDia2-FH2 with homology arms complementary to pCDH vector. |
| mDia2-FH1-FH2-R | taagcttggtaccgaggatccAGTTTTCATTTCTAGTAAGCGCTTTTT |  |
| mDia2-DAD | | |
| mDia2-DAD-F | ggatctatttccggtgaattcGCCACCATGGAGGGAGATG AGACA | Forward and reverse primers for PCR amplification of mDia2-DAD with homology arms complementary to pCDH vector. |
| mDia2-DAD-R | taagcttggtaccgaggatccTAAAGCTCGTAATCTTGCCAGCA |  |
| mDia1 FL | | |
| mDia1-FL-F | gttccagattacgcttctagaATGGAGCCGTCCGGCGGG | Forward and reverse primers for PCR amplification of mDia1 full length with homology arms complementary to the HA sequence at 5’ and to pLVX-Neo vector at 3’. |
| mDia1-DAD-R | gggctcgaggttaacggatccTTAGCTTGCACGGCCAACC |  |
| mDia1-GBD-DID | | |
| HA-mDia1-GBD-DID-F | ggatctatttccggtgaattcGCCACCATGTACCCATACGATG | Forward and reverse primers for PCR amplification of HA tagged mDia1-GBD-DID with homology arms complementary to the modified pLVX-Neo vector. |
| HA-mDia1-GBD-DID-R | gggctcgaggttaacggatccTTAGCTAGAAACAGAAGGTGCAACA |  |
| mDia1-DID | | |
| mDia1-DID-F | gttccagattacgcttctagaCCGCAGCCGGAGGACATG | Forward and reverse primers for PCR amplification of mDia1-DID with homology arms complementary to pLVX-Neo vector with HA tag. |
| mDia1-DID-R | gggctcgaggttaacggatccTTAGCTAGAAACAGAAGGTGCAACA |  |
| mDia1-FH1-FH2 | | |
| mDia1-FH1-FH2-F | gttccagattacgcttctagaAGTGCTGCTGTTCCCCCTG | Forward and reverse primers for PCR amplification of mDia1-FH1-FH2 with homology arms complementary to pLVX-Neo vector with HA tag. |
| mDia1-FH1-FH2-R | gggctcgaggttaacggatccTTACACACCTGTCTCATCCCCC |  |
| mDia1-FH1-FH2-DAD | | |
| mDia1-FH1-FH2-F | gttccagattacgcttctagaAGTGCTGCTGTTCCCCCTG | Forward and reverse primers for PCR amplification of mDia1-FHDAD with homology arms complementary to pLVX-Neo vector with HA tag. |
| mDia1-DAD-R | gggctcgaggttaacggatccTTAGCTTGCACGGCCAACC |  |
| mDia1-ΔDAD |  |  |
| mDia1-FL-F | gttccagattacgcttctagaATGGAGCCGTCCGGCGGG | Forward and reverse primers for PCR amplification of mDia1-ΔDAD with homology arms complementary to the vector. |
| mDia1-FH1-FH2-R | gggctcgaggttaacggatccTTACACACCTGTCTCATCCCCC |  |
| FHL2 | | |
| FHL2-F | GCAACCGGTATGACTGAACGCTTTGACT | Forward and reverse oligoes for PCR amplification of murine FHL2, conventional ligation was used for inserting the FHL2 CDS into MI-tagRFP657. |
| FHL2-R | GCCGTCGACTCAAATATCCTTTCCACAGTCA |  |
| 3×SRE Luciferase construct | | |
| SRE-LUC-F | C ccctatatggccttatatggccatatatggccaaataagg A | Forward and reverse oligoes for inserting artificial quadruple SRE sequence into pGL4.22 between 5’ *KpnI* and 3’ *BglII* sites. |
| SRE-LUC-R | GATCTccttatttggccatatatggccatataaggccatataggg GGTAC |  |
| pET28a-mDia1 FL |  |  |
| His-mDia1-FL-F | ATGGGTCGCGGATCCGAATTCATGGAGCCGTCCGGCGGG | Forward and reverse primers for PCR amplification of mDia1-FL with homology arms complementary to pET28a vector. |
| His-mDia1-FL-R | GTGGTGGTGGTGGTGCTCGAGGCTTGCACGGCCAACCAG |  |
| pET28a-mDia1-GBD-DID | | |
| His-mDia1-GBD-DID-F | AAAGAATTCATGGAGCCGTCCGGCGG | Forward and reverse primers for PCR amplification of mDia1-GBD-DID, conventional ligation was used for vector construction. |
| His-mDia1-GBD-DID-R | TGGTGCTCGAGGCTAGAAACAGAAGGTG |  |
| pGEX-4T-1-mDia2 FL | | |
| GST-mDia2-FL-F | ATCCCCGGAATTCATGGAGAGGCACCGG | Forward and reverse primers for PCR amplification of mDia2-FL with homology arms complementary to the pGEX-4T-1 vector. |
| GST-mDia2-FL-R | CGATGCGGCCGCTCGAGTTATAAAGCTCGTAAT |  |
| pGEX-4T-1-mDia2-DAD | | |
| GST-mDia2-DAD-F | CCGGAATTCGAGGGAGATGAGACAGGAGT | Forward and reverse primers for PCR amplification of mDia2-DAD, conventional ligation was used for vector construction. |
| GST-mDia2-DAD-R | GCCGCTCGAGTTATAAAGCTCGT |  |
| pGEX-4T-1-mDia2-FH1-FH2 | | |
| GST-mDia2-FH1-FH2-F | CCCCGGAATTCTTGCCACCTGGTACAA | Forward and reverse primers for PCR amplification of mDia2-FH1-FH2, conventional ligation was used for vector construction. |
| GST-mDia2-FH1-FH2-R | GCGGCCGCTCGAGTTAAGTTTTCATTTCTAGTAAGCGC |  |
