# Supplementary material for: mDia formins form hetero-oligomers and cooperatively maintain murine hematopoiesis
Source: PLoS Genet. 2023 Dec 29;19(12):e1011084. doi: 10.1371/journal.pgen.1011084 (PMC10756686; doi:10.1371/journal.pgen.1011084)
Supplement: S2 Table — (DOCX) [file pgen.1011084.s011.docx]

**S2 Table.** Primer sequences for real-time quantitative PCR.

| **Target Genes** | **Sequence (5’ > 3’)** | **Notes** |
| --- | --- | --- |
| *mDia1* | F: TCCAAGCTGACAGGAGAGGT | Forward and reverse primers for qPCR detecting murine *mDia1*. |
|  | R: GGGGGAGGTGGAATAACAGT |  |
| *mDia2* | F: AGCCTTGACTTCAGCTGGAG | Forward and reverse primers for qPCR detecting murine *mDia2*. |
|  | R: GGTGAAGCCTGAAGTCCAAA |  |
| *hDIAPH1* | F: GTTCTGCCATTTGGATTAACC | Forward and reverse primers for qPCR detecting human *mDia1*. |
|  | R: ACATGCAGCAGTGACAGACAC |  |
| *hDIAPH3* | F: TGAGTGGCCACCTTCTTT | Forward and reverse primers for qPCR detecting human *mDia2*. |
|  | R: TTGTCCAGCATATCATCCGTC |  |
| *18s rRNA* | F: GCAATTATTCCCCATGAACG | Forward and reverse primers for qPCR detecting both human and murine 18s rRNA, and used as internal control. |
|  | R: GGCCTCACTAAACCATCCAA |  |
| *The quantitative PCR primers for detecting *KRT17*, *Acta2*, *Flna*, *FHL2*, *ITGAL*, *c-Fos*, *ITGA2*, *Egr1*, *Egr2*, *Egr3*, *Fosb* and *JunB* have been described elsewhere[1]. | | |

Supplemental references:

1. Mei, Y., et al., *Diaphanous-related formin mDia2 regulates beta2 integrins to control hematopoietic stem and progenitor cell engraftment.* Nat Commun, 2020. **11**(1): p. 3172.
